# Immunopathology of Recurrent Vulvovaginal Infections: New Aspects and Research Directions

**DOI:** 10.3389/fimmu.2019.02034

**Published:** 2019-08-28

**Authors:** Namarta Kalia, Jatinder Singh, Manpreet Kaur

**Affiliations:** ^1^Department of Molecular Biology and Biochemistry, Guru Nanak Dev University, Amritsar, India; ^2^Department of Human Genetics, Guru Nanak Dev University, Amritsar, India

**Keywords:** adaptive immunity, innate immunity, oxidative stress, evasion, single nucleotide polymorphisms (SNPs), pattern recognition receptors (PRRs), infectious diseases

## Abstract

Recurrent vulvovaginal infections (RVVI), a devastating group of mucosal infection, are severely affecting women's quality of life. Our understanding of the vaginal defense mechanisms have broadened recently with studies uncovering the inflammatory nature of bacterial vaginosis, inflammatory responses against novel virulence factors, innate Type 17 cells/IL-17 axis, neutrophils mediated killing of pathogens by a novel mechanism, and oxidative stress during vaginal infections. However, the pathogens have fine mechanisms to subvert or manipulate the host immune responses, hijack them and use them for their own advantage. The odds of hijacking increases, due to impaired immune responses, the net magnitude of which is the result of numerous genetic variations, present in multiple host genes, detailed in this review. Thus, by underlining the role of the host immune responses in disease etiology, modern research has clarified a major hypothesis shift in the pathophilosophy of RVVI. This knowledge can further be used to develop efficient immune-based diagnosis and treatment strategies for this enigmatic disease conditions. As for instance, plasma-derived MBL replacement, adoptive T-cell, and antibody-based therapies have been reported to be safe and efficacious in infectious diseases. Therefore, these emerging immune-therapies could possibly be the future therapeutic options for RVVI.

## Introduction

Vulvovaginal infections (VVI) are the commonly reported microbiological syndrome affecting millions of women globally in all strata of society. An abnormal vaginal discharge is the key trait and first sign of VVI that women seeking health care frequently complaint to gynecologist. About a quarter of all adult women complain about abnormal vaginal discharge, more commonly by those belonging to Indian subcontinent i.e., South Asia (1). Moreover, the repeated experiences of common vaginal infections collectively known as recurrent VVI (RVVI) are emerging and are the major concern for researcher these days. The three common RVVI are Bacterial Vaginosis (BV), Vulvovaginal Candidiasis (VVC), and Trichomoniasis (TV) (2). The recurrence rate of BV (RBV) is as high as 30–50% within 3 months while ≥ 4 repetitive episodes of VVC in 12-months are referred as recurrent VVC (RVVC) (3). Similarly, cases of recurring TV (RTV) have also been reported with recurrence rates as high as 5–8% within 2 months of initial diagnosis (4). The milieu conditions of vagina during one VVI type create a niche for the pathogenesis of other VVI, leading to Mixed Infections (MI) and co-infections (5, 6). These VVI, left untreated, will not only affect the female reproductive health, but may also result in many foster infections/diseases and adverse pregnancy outcomes (7–9).

The literature regarding RVVI pathogenesis has suggested that “a vaginal microbiota (VMB) dominated with *Lactobacilli* is healthier than a diverse VMB.” This diversity in VMB causes dysbiosis characterized by fall in number of *Lactobacilli* and overgrowth of opportunistic pathogens that are either normally present in human VMB in lower quantity or sexually transmitted, resulting in RVVI (10). However, the decades of research failed to find a single pathogen responsible for causing common RVVI. This is because 20–30% of healthy (asymptomatic) women were found to have VMB same as that of VVI women (11). Also, some *Lactobacilli* strains, i.e., *L. iners* and *L. jensenii*, were found to be pathogenic and associated with VMB instability in pregnant women, pre-term birth, and BV (12, 13). Therefore, the present scenario doesn't satisfy the Koch's postulates, according to which the causative agent is essential for causing disease and it should not be present in population without disease (14). Due to this reason, accurate definition of vaginal health and vaginal infections is still lacking till date. Besides this, there are other local risk factors that have also been suggested to create favorable conditions for the development of RVVI (15). However, development of RVVI in women lacking any of these recognized disposing factors, suggests the involvement of host immune components, instrumental in elimination of RVVI pathogens (16). Further suggesting that, it's the host immune system that determines the disease outcome and thus must be explored to get an accurate definition of vaginal health and infections.

Thus, an attempt has been made, to achieve a clear understanding of host immunity in three common RVVI. This review commences with a brief summary of what is known about immunology of human vagina. Different studies that contributed to host defenses against common RVVI were then addressed and linked. These protective immune mechanisms lead to oxidative stress that has been shown to play a major role in pathophysiology of common RVVI. The review further fine points the hijacking or exploitation of host immune responses by RVVI pathogens. A theory and related mechanism was then proposed to explain the immunopathogenesis of RVVI. Moreover, genetic variations in immune molecules have been shown to play an important role in how a woman responds to a particular RVVI challenge, as evidenced by several genetic disease association studies that are detailed in this review. Based on this comprehensive compilation, different strategies were proposed for treatment of RVVI that may prevent recurrence of these enigmatic infections.

## Immunology of Human Vagina

The human vagina consists of multiple levels of protection in form of innate and adaptive immunity that is further compartmentalize into various components and is under strong hormonal control. Inflammation signifies an essential immune mechanism that is meant to eliminate pathogens and repair damage caused by deleterious stimuli. Theoretically, inflammation is a process that involves four stages, including an activating system, a sensing mechanism, signal diffusion, and the effector cells activation (17). In infectious diseases, the activating system is pathogen that has preserved biomolecular structures on its surface known as pathogen associated molecular patterns (PAMPs). These PAMPs lead to the activation of quick and non-specific innate immunity that further signals for the activation of specific adaptive immunity, with the ultimate goal of eradicating the pathogens and repairing tissue damage elicited by the noxious stimuli (17).

### Innate Immunity in Human Vagina

The innate immuny of human vagina involve physical, chemical, and cellular components (18). Interactions between these components form a complex microenvironment that mediates immune responses in vagina, regulated by sex hormones and specific microbiome (19).

#### Physical Barriers

Mucosal lining and epithelial cells serve as gatekeepers preventing the entry of pathogens in to vagina (20). The gel like mucosal layer is produced by the mucin proteins expressed by the surface of upper layer of epithelial cells throughout the vagina (21). Besides entrapping the invasive pathogens, the vaginal mucosal layer also provides lubrication and acts as a source of nutrition for the VMB. In turn, the VMB of healthy women (dominated by *Lactobacilli*) contributes to the physical defense of vaginal mucosa against pathogens by maintaining low pH, producing lactic acid, and other antimicrobial substances (22). Thus, VMB is capable of modulating defense property of vaginal mucosa. However, many physiological processes including menstruation, conception, pregnancy, and the hormonal changes frequently modulate the vaginal mucosal immune system (23). The stratified squamous vaginal epithelium, underlying the mucosal layer, also acts as a barrier and first responder to pathogens by “sensing” the danger leading to immune cell activation and secretion of immune mediators driving inflammation and immune responses (24). The identified danger is the damage done to vaginal epithelial cells by virulence factors secreted by the pathogens. These damaged host cells derived immune mediators are called damage-associated molecular patterns (DAMPs, danger signals, or alarmins) that constitute the parts of chemical components of immune system. Beneath the epithelium is lamina propria that is composed primarily of fibroblasts, blood vessels, and a diversity of immune cells (explained under cellular components).

#### Chemical Components

DAMPs, PRRs, chemotactic cytokines, AMPs, and C system make the chemical components of innate immune system of vagina. Other than injured host cells, DAMPs are also released under conditions like necrosis, apoptosis, and by collapsed extracellular matrix (25). Some examples of intracellular DAMPs includes DNA, fibronectin, high mobility group box-1 (HMGB1), S-100 proteins, heat shock proteins, hyaluronic acid, formyl peptides, ATP, and collagen or elastin derived peptides (25). In order to discriminate own cells from pathogens, the vaginal immune system employ pattern recognition receptors (PRRs), that specifically respond to various pathogens (26). Currently, PRRs are divided into five major families including Toll-like receptors (TLRs), C-type lectin receptors (CLRs), the nucleotide-binding oligomerization domain (NOD) like receptors (NLRs), retinoic acid-inducible gene (RIG) I-like receptors (RLRs), and absent in melanoma 2 (AIM2)-like receptors (ALRs) (27). The PRRs expressed by squamous vaginal epithelial cells include TLRs i.e., TLR1-10 except TLR7, CLRs including dendritic cell-associated c-type lectin-1 (Dectin-1) and secretary mannose binding lectin (MBL) as well as NOD receptor including NOD1 (18, 28–32). The expression of TLR4 and Dectin-1 in the vaginal epithelial cells is controversial as some studies have shown their presence, while others reported their absence (28, 33, 34). Also, the MBL levels in vaginal fluid are partly contributed from plasma, as a result of transudation, though directly secreted by local vaginal epithelial cells (32). However, almost all the known human TLRs, CLRs and intracellular PRRs are expressed by immune cells of both myeloid (neutrophils, macrophages and dendritic cells) and lymphoid origin (B and T cells) present in lamina propria or in vagina due to transmigration (29, 30). Upon stimulation, either through direct contact of PAMPs or through indirect means (DAMPs or cytokines), these PRRs initiates a signaling cascade, that includes activation of transcription factors, release of antimicrobial peptides (AMPs) as well as chemotactic cytokines that further signals for the activation of adaptive immunity and subsequent amplification of innate immune responses, leading to the ultimate killing of pathogens (35).

AMPs are generally expressed by numerous cell types of vagina primarily by neutrophils and epithelial cells, with small fractions contributed by dendritic cells (DCs), macrophages, and natural killer (NK) cells (36). These AMPs have anti-microbial properties against bacteria, fungus, parasite, and virus (23, 36). Different AMPs that have been reported in lower genital tract include defensins, protease inhibitors, including serine protease inhibitors (serpins), secretory leukocyte protease inhibitor (SLPI), human epididymis protein 4 (HE4), cystatins, elafins, lysozyme, lactoferrin, and cathelicidin (LL-37). Out of these, human defensins [both alpha (α) and beta (β)] are among the most widely characterized and abundant AMPs present in lower genital tract including vagina (18, 23, 37). Besides antimicrobial properties, these AMPs can destroy target cells through modulating pH and ionic concentration gradient and also have been shown to have chemotactic activity (36). The detailed mechanisms of action for each AMP have been comprehensively reviewed elsewhere (36, 38, 39). As mentioned, all the immune and vaginal epithelial cells upon activation release chemical messengers called cytokines that create an aggressive milieu for the pathogen either by creating a network between the different immune cell types or by providing direct antimicrobial response (20). Different cytokines are released based on different stimuli and cell type. Other than this, the complement (C) system, a humoral component of the innate immune system, is actively involved in the host protection against vaginal infections (40). The C system recognizes the pathogens by utilizing three different molecules i.e., C1q, MBL (also a CLR) and C3, that respectively trigger the classical, lectin and alternative pathways for pathogen elimination (41).

#### Cellular Components

Inflammatory immune cells e.g., Neutrophils, Macrophages, NK cells, and DCs, which are either resident (like epithelial cells) or transmigrated into the genital tract in response to DAMPs or chemotactic cytokines, form the cellular components of innate immune system (42). Neutrophils are the major cells that are recruited at the site of infection, mediating an inflammatory response against pathogens. Neutrophils are present throughout the female reproductive tract with pre-dominant number in fallopian tubes. However, the quantity steadily reduced from the upper genital tract to vagina (23, 42). However, upon infection, under the influence of chemotactic cytokines e.g., IL-8, abundant amounts of neutrophils penetrate from vaginal epithelium into the lumen to phagocytise the pathogens and cellular debris (43). Furthermore, the proportion of these neutrophils also increases prior to menses, due to the natural process of tissue breakdown, and during copulation for the phagocytosis of sperm (18). Other than phagocytosis, these neutrophils also respond to pathogens through production of oxidative compounds, leading to oxidative stress, by releasing AMPs and cytokines for its own stimulation or for the recruitment of other cells. Other important innate immune phagocytic cells include macrophages, DCs and NK cells that constitute 10% of the total leukocytes present in the female genital tract (18, 44). Macrophages and DCs act as professional antigen presenting cells (APCs) for inducing of adaptive immune responses (45). Moreover, only DCs in particular act as a main mediator for bridging the innate and adaptive immunity and generate life-long memory by priming naive T-cells (46). In addition, NK cells in vagina lead to macrophage activation and generate pro-inflammatory and cytotoxic T cell responses (18, 20).

### Adaptive Immunity of Human Vagina

The adaptive immune system of female reproductive tract presents distinctive characteristics that are unique from the adaptive immunity of other mucosal surfaces (18). It involves immunoglobulins and various cellular components (18, 47).

#### Chemical Components

Different studies have documented antibody (the humoral component of adaptive immunity) responses against vaginal infections based on *in vivo* or *in vitro* experiments and suggested weak but consistent presence of IgG and IgA antibodies in vaginal secretions (47). Although both antibodies are present in genital secretions, IgG was found to be more predominant than IgA (48). These antibodies prevent colonization of pathogen by checking their adherence to vaginal epithelial cells and contribute to the neutralization and formation of Ag-Ab complexes, helping in uptake and clearance of pathogen by phagocytic cells of vagina (47, 48).

#### Cellular Components

The cellular components of vaginal adaptive immunity include effector B-cell, CD4^+^, and CD8^+^ T cells responses as well as local B and T memory cells that are found throughout the female reproductive tract. The T cells in vaginal tissue are localized at the stroma/epithelial interface and are few in number (49, 50). Moreover, recent studies have also suggested the presence of T-helper 17 (Th17) cells and regulatory T (Treg) cells in vagina (51). Other than this, various immuno-histochemical studies have shown the presence of antibody-producing B cells with low prevalence in vagina, ectocervix, and fallopian tubes relative to endocervix (47). However, during inflammation the number of intra-epithelial lymphocyte population increases relative to non-inflamed vagina.

## Immunological Host Defenses Against Common RVVI

To maintain homeostasis and minimize the risk of infection, host vagina is capable and competitive enough to generate different immune responses against different vaginal infections as given in detail below:

### Immunity in BV

**Figure 1:**
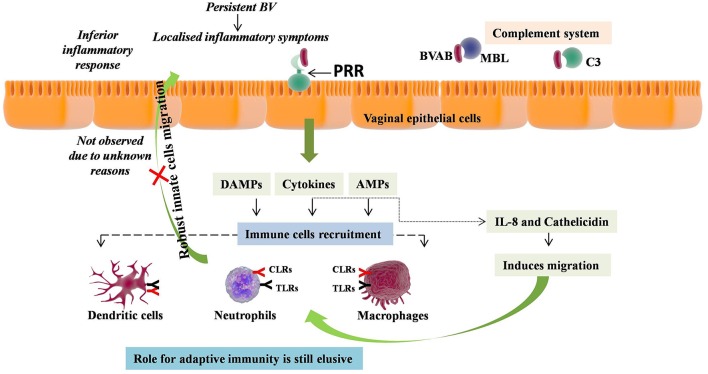
Immunopathology of Bacterial Vaginosis (BV). BV induces milieu enriched with proinflammatory cytokines and antimicrobial peptides (AMPs) in vagina. This enriched vaginal milieu has been induced by recognition of BV associated bacteria (BVAB) by pattern recognition receptors (PRR) such as the TLR4, MBL, and C3. The role of these cytokines and AMPs is to induce migration of neutrophils, macrophages, and monocytes. However, no such migration has been reported in BV, depicting the inferior inflammatory response without any localized inflammatory symptoms. Conversely, persistent BV has been shown to lead chronic and extreme vaginal inflammation, while the role of adaptive immunity is still elusive.

Studies based on transcriptional profiling and markers assessment in vaginal secretions and serum has indicated the major involvement of host immunity in BV (Figure 1). Assessment of vaginal secretions in BV women has shown the stimulation of nuclear factor-κB (NF-κB) in various cell types, which is the characteristic factor involved in proinflammatory signaling pathways of many TLRs (52). However, the major TLR found to be involved in BV pathology is TLR4, whose expression in monocytes is shown to be strikingly increased on exposure to lavage samples of BV women (53). Moreover, it was shown that sensing of BV associated bacteria is facilitated *in situ via* TLR4 signaling, through NF-κB pathway leading to lymphocytes enrolment by cytokines secretion, thus causing genital inflammation (54). Other than this, immunofluorescence analysis of clue cells from BV patients revealed the presence of MBL and C3 on clue cells suggesting their direct role in recognition of BV associated bacteria (BVAB) and activation of both lectin and alternative pathways of complement system (40). Complementry evidence recommended that the chances of acquiring BV will be more in cases with insufficient sMBL levels (55). Expression of Dectin-1, another PRR, was found to increase upon stimulation with bacterial LPS relative to primarily observed low expression in freshly isolated human peripheral blood monocytes (PBMCs) and human monocytes cell line detected by both qPCR for mRNA and FACS staining for cell surface protein expression (56). In consonance, high serum Dectin-1 levels were observed in BV patients relative to controls, suggesting the active role played by Dectin-1 in defense eagainst BV (57).

In addition, cytokines have been shown to directly contribute to BV pathology, as, BV was found to induce milieu enriched with proinflammatory cytokines in the lower genital tract. These include IL-2, IL-4, IL-6, IL-8, IL-10, IL-12p70, IL-1β, IL-1α, TNF-α, IFN-γ, FMS-like tyrosine kinase 3 ligand, chemokine C-C motif ligand 5 (CCL5), and SLPI [compiled in 58 and currently reported in Jespers et al. (58) and Lennard et al. (59)]. These studies supported the concept that BV triggers the innate immune responses, which was also assessed independently for individual bacterial species present in VMB of BV patients. For example, *A. vaginae* was found to induce expression of IL-1β, IL-6, IL-8, CCL20, human β defensin-2 (HBD-2), and TNF-α via NF-κB, TLR2, and MyD88 signaling pathways; *G. vaginalis* was found to induces IL-1β, IL-18, IL-6, IL-8, and TNF-α; *Mobiluncis curtisii* and *Prevotella bivia* induced IL6, IL8, G-CSF, IP-10, MIP-1β, RANTES, and Gro-α (60–64).

Of all the associated pro-inflammatory cytokines, IL-1β was consistently been linked with BV, with four to over 10-fold higher levels than controls, validated both by earlier as well as current studies (58, 65–75). Moreover, successful treatment of BV has been shown to normalize the elevated pro-inflammatory cytokines levels (76). IL-1β, a pro-inflammatory cytokine produced by innate immune cells, is a key mediator of the inflammatory response and is essential for the host-response and resistance to pathogens (77). IL-1β has a special role in the altering production of pro-inflammatory cytokines such as IL-8, IL-6, and TNF-α and found to be positively correlated with their levels and cell surface expression of TLR4 in human epithelial cells (78). This suggests that the secondary pro-inflammatory cytokines should also increase with increase in IL-1β levels during BV. This was further confirmed by studies that have found higher levels of IL-8 in women with BV (67, 72). In contrast, inconsistent but elevated expression of IL-1α was also found to be associated with BV (65, 79, 80).

Other than cytokines, AMPs, particularly defensins, were found to be associated with BV, though their role in pathogenesis is not clear due to inconsistencies in the results obtained. Elevated expression of human α-defensins, mainly formed by neutrophils and epithelial cells, were observed in BV with intermediate flora (81). Furthermore, a study has also shown increased expression of HBD-2 in vaginal epithelial cells elicited by BVAB, while no such association was found by another study (63, 82). Expression of other AMPs including SLPI and HE4 was also found to be associated with BV organisms, but their negative association were also reported (63, 83). The high levels of AMPs, including S100A8 and calprotectin, were found in lavage samples of BV cases (84). These proteins bind manganese, leading to decrease in availability of free manganese which is an absolute requirement for *lactobacilli* to grow, thus inhibiting their proliferation and creating milieu for BVAB to grow (85). Another AMP i.e., cathelicidin was also observed at high levels in BV women, acting as a pore-forming toxin to disrupt membrane of pathogenic bacteria (86, 87). Cathelicidin also induces the migration of neutrophils, macrophages, and monocytes, ultimately leading to inflammatory response against infection (88). Recently, high levels of lactoferrin, an iron binding AMP, which is predominantly produced by neutrophils, were also reported in BV women (89). Iron sequestering ability of lactoferrin leads to depletion of free iron that is required for the growth by BVAB. The elevated levels of α-defensins, lactoferrin, and cathelicidin in BV suggest the possibility of high neutrophils levels in BV, however, no such elevation has been observed (69, 90).

On the other hand, DCs maturation has been shown to be induced by lavage samples from women with BV leading to DC-surface expression of CD83 and CD86 markers, reduced internalization ability, improved antigen presentation to T cells, thereby modulating T cell responses (91). The same profile of activation and maturation of DCs and T cells has been reported recently but found to occur only at the highest *G. vaginalis* concentration (92). No heightened neutrophils levels and maturation of DCs only at high bacterial concentration depicts the uncharacteristic and poor inflammatory response without any localized inflammatory symptoms, perhaps the reason why BV is not called bacterial vaginitis. However, very recently a longitudinal study has shown occurrence of chronic and extreme vaginal inflammation due to persistent BV (59). Overall, these studies confirm the inflammatory nature of BV. Absence of local inflammatory responses can be attributed to evasion of BVAB or perhaps the presence of genetic polymorphisms in inflammatory genes modifying the inflammatory responses.

### Immunity in VVC

Unlike BV, where the host immediately recognize and generate immune responses against BV associated bacteria, which are the different entities from the normally present *Lactobacilli*, VVC involves the same entity *Candida*, present both as commensal as well as responsible for pathogenesis. The pathogenic activity of *Candida* is determined by its morphology, where the yeast form is associated with commensalism, and hyphal form with pathogenicity. The detection and elimination of *Candida* pathogenic form is mediated by vaginal epithelial cells, the first barrier encountered by pathogen (93). Vaginal epithelial cells “sense” the danger constituted by the pathogen and respond by immune cell activation, secretion of inflammatory immune mediators and by generating immune responses. The danger can be attributed to virulence factors secreted by *Candida* hyphae e.g., secreted aspartic proteases (Saps). Saps can directly lead to neutrophils recruitment at the site of infection (94). However, a new protease, namely Candidalysin, has recently been proposed to be secreted by *Candida* hyphae (95). This protease is the first protein toxin recognized in any human fungal pathogen that damages the epithelial cells and thus triggers host immune responses. Candidalysin has recently been proposed as a key hypha-associated virulence factor responsible for immunopathogenesis of VVC (96). By sensing candidalysin activity, vaginal epithelial cells respond to pathogenic *C. albicans* through activation of two signaling pathways i.e., p38/c-Fos and MKP1 pathways (95, 96). The same pathways have been suggested as common mechanism facilitating different human epithelial cells for differentiating pathogenic *Candida* from non-pathogenic yeast form and to coordinate innate immune responses (93, 97). The receptors employed by epithelial cells to sense candidalysin activity are still pending to be eludicated. The resulted activation of p38/c-Fos and MKP1 signaling pathways persuades expression of pro-inflammatory cytokines and AMPs including IL-1α, IL-1β, IL-8, G-CSF, GM-CSF, β-defensin 3, CCL20, S100A8, and S100A9 from vaginal epithelial cells, which are instrumental for innate immune cells recruitment (95–97). Vaginal epithelial cells were shown to produce calcium-binding proteins namely S100A8 and S100A9 in response to *C. albicans* that lead to robust neutrophils migration during VVC (98, 99). However, their involvement was not found to be crucial for driving the neutrophils response in VVC (100).

Neutrophils and macrophages are the first innate immune cells that recruit at the site of infection in response to affected epithelial cells derived immune mediators. The neutrophils further release TNF-α that consequently up-regulates TLR4 expression on epithelial cells (101). Independent to these virulence factors and cytokines response, direct contact mediated recognition of *C. albicans* sugar moieties such as mannan and β-glucan is mediated by neutrophils, marcrophages, and dendritic cells via surface PRRs including TLRs (including TLR 2, 4, and 9) and CLRs (including MBL, Dectin-1, Dectin-2, DC-SIGN, and Mincle) (40, 102, 103). Ligation of PRRs further stimulate downstream MAPK and Syk signaling, production of NF-kB, and pro-inflammatory cytokines. The activated immune cells form a loop of positive feedback that amplifies the inflammatory process, leading to ultimate effectors functions. Other than this, immunofluorescence analysis of the *Candida* hyphae from VVC patients showed the presence of C3 and pH dependent binding of MBL suggesting their direct role in *Candida* recognition and activation of alternative as well as lectin complement pathways (40, 102). In addition, MBL have been shown to cause agglutination of *Candida* upon hyphae generation independent of direct/indirect opsonophagocytosis and complement activation (104). In consonance, two different studies have reported high MBL levels in women with VVC than healthy women, suggesting the active role of MBL in the defense against VVC (105, 106). Other complementary evidences have shown that low MBL levels in women predispose them to VVC (55, 107, 108).

Moreover, neutrophils, through direct contact via PRRs particularly CLRs, mediate killing of *Candida* with short hyphae intracellularly and those with long hyphae extracellularly. The intracellular killing is mediated by phagocytosis while, extracellular killing is mediated by NETosis i.e., development of neutrophils extracellular traps (NETs). Both mechanisms involve oxidative burst due to reactive oxygen species (ROS) production (109–113). Additionally, autophagy, fibronectin, release of granular enzymes, AMPs including calprotectin and Dectin-1 signaling has also been shown to be involved in NETosis (109, 110, 112, 114, 115). In contrast, some other studies suggested that β-glucan mediated NETosis occurs through complement receptor 3 (CD11b/CD18) and not through Dectin-1 signaling or ROS mechanism, indicating their controversial role in NETosis (111, 114). Furthermore, recruitment of polymorphonuclear neutrophils (PMN) into vagina was observed to be associated with vaginal inflammatory symptoms when volunteer women were challenged with live *C. albicans* (116). This vaginal inflammation was observed to decrease with diminution of PMNs (117, 118). Robust response by neutrophils recruitment correlates well with local vaginal inflammation similar to high vaginal *Candida* burden for causing VVC. However, a mechanism explaining PMN dysfunction at the vaginal mucosa remained a mystery. The most current information relative the mechanism(s) of the VVC immunopathogensis where a strong inflammatory condition occurs (via candidialysin and hyphal morphology transition), but fail to reduce the *Candida* load and thus the persistence of infections has been proposed (119). This has recently been termed as “neutrophil anergy” and involves vaginal factors that inhibit the ability of the PMN to bind to *Candida* for effective killing (119). Unlike neutrophils, macrophages mediate only intracellular killing of *Candida* but both through ROS and RNS mechanisms (120).

Just like surface PRRs, the intracellular PRR i.e., NLRs of innate immune cells are also activated by DAMPs or through direct contact of internalized pathogenic fungal components that activate NLRP3 inflammasome, consequently leading to release of proinflammatory cytokines including IL-1β and IL-18 (121). This inflammasome activation also led to programmed host cell death known as pyroptosis, which is employed as one of the evasion strategies by virulent *Candida* for escaping macrophages (122, 123). Owing to this reason, the macrophages mediated killing of intracellular *C. albicans* is shown to be of lower efficiency than neutrophils (103, 121). However, the fungal triggers activating the inflammasome mediated pyroptosis are still not known. Finally, IL-1β and IL-18 release through inflammasome activation by innate immune cells promote adaptive T helper 17 (Th17) and Th1 responses respectively, thus linking innate with adaptive immunity (124). Moreover, both *in vitro* and *in vivo* studies have shown that Dectin-1-Syk-CARD9 signaling couple innate and adaptive immunity independently of TLR signals and induce differentiation of adaptive Th-17 and Th-1 cells (125, 126).

Carvalho and group showed that Dectin-1 is necessary for controlling vaginal infections (127). The study evaluated two genetically distinct strains of mice with vaginal candidiasis for Dectin-1 deficiency and showed that the role of Dectin-1 in antifungal immunity lies ahead of Th17 cell activation and is significantly dependent on host genetic milieu. The study found that Dectin-1 was required for appropriate control of vaginal candidiasis in mice strain C57BL/6, but not in BALB/c mice. The former Dectin-1 deficient strain of mice was found to be vulnerable to infection, with defective production of cytokines including IL-17A as well as IL-22 and adaptive Th1 responses, relative to reverse effect observed in latter strain. However, this depiction of two tremendously contradictory phenotypes, have been attributed to differential expression of functionaly distinct Dectin-1 isoforms by two strains (128). Thus, the study clearly depicts that Dectin-1 essentially contributes to the stability of Th1, Th17, Treg CD4^+^ T-cell populations during infection, as its deficiency lead to defective release of Th17 cells in C57BL/6 mice and both Th1 as well as Treg cells in BALB/c mice after infection. This suggests the contribution of Dectin-1 in differentiation of T helper cells and its relative ability to control the vaginal infection. In consonance to this, another study highlighted the specific role of Dectin-1 in VVC in four women from Netherlands, affected either by onychomycosis or RVVC (129). The Dectin-1 expression in these women was found to be poor with defective β-glucan binding, defective Th17 responses, and defective production of cytokines including IL-6, TNF-α, and IL-17. Another study has shown increased intracellular expression of Dectin-1 in response to opsonised *Candida albicans* through recognition of β-1,3-glucan (130). In consonance, a recent study found significantly high serum Dectin-1 levels in VVC cases relative to controls suggesting the active role played by Dectin-1 in defense against VVC (57). Thus, Dectin-1 is certainly a formidable PRR for providing systemic immune defense against the infection however, its role as a mucosal defense marker is still ambiguous due to inconclusive literature regarding its normal expression on vaginal mucosa as aforementioned. Therefore, caution should be taken regarding these interpretations. Moreover, two studies based on different animal models (rat and mouse) of VVC, have depicted the controversial role of dendritic cells following infection with no definite conclusion (131, 132).

**Figure 2:**
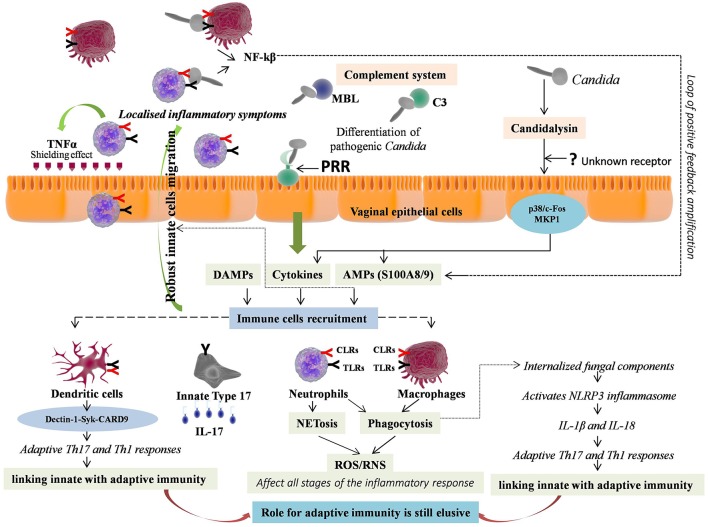
Immunopathology of vulvovaginal candidiasis (VVC). Vaginal epithelial cells respond to pathogenic form of *Candida* either through direct contact via Pattern recognition receptors (PRRs) or by sensing hypha associated virulence factor e.g., Candidalysin, through unknown receptor, further activating two signaling pathways i.e., p38/c-Fos and MKP1. The response includes immune cell activation, secretion of inflammatory immune mediators, which are instrumental for innate immune cells recruitment. Neutrophils and macrophages are the first to recruit at the site of infection. The neutrophils release TNF-α that provides shield from infection by up-regulating TLR4 expression on epithelial cells. The expressed PRRs on ligation with pathogen further stimulate downstream signaling, production of NF-kB and pro-inflammatory cytokines, forming a loop of positive feedback. The effector cells mediate killing of *Candida* with short hyphae by phagocytosis and those with long hyphae by NETosis. Both mechanisms involve reactive oxygen species (ROS) production that further regulates all the stages of inflammation. The phagocytised pathogenic components activate NLRP3 inflammasome through intracellular NLRs, and lead pro-inflammatory cytokines release including IL-1β and IL-18, which further promote adaptive T helper 17 (Th17) and Th1 responses respectively, thus linking innate with adaptive immunity. Independently of this, Dectin-1-Syk-CARD9 signaling also couple innate and adaptive immunity and induce differentiation of adaptive Th-17 and Th-1 cells. The activated Th17 cells release IL-17, which further increases expression of PMNs, chemotactic cytokines and AMPs, promoting extreme vaginal inflammation. However, an array of innate immune cells called “innate Type 17” cells also produces IL-17. Thus, valuable information relative to adaptive T cell-mediated immunity (CMI) remained elusive in VVC.

The activated Th17 cells release IL-17, a multifunctional pro-inflammatory cytokine, that further increases expression of PMNs, chemotactic cytokines and AMPs (HBD-2/3, histatins), promoting effective inflammatory response (133–135). Studies have shown that inhibition of Th17 cells differentiation led to considerable decrease in production of IL-17 and HBD-2 with consequent exacerbation of VVC (136–138). However, some studies have suggested that inflammatory response during VVC occurs independently of Th17 cell lineage (99, 139, 140). Hence, valuable information relative to adaptive T cell-mediated immunity (CMI) remained elusive as almost equal number of studies are recommending and contrasting its role in VVC. Additionally, studies have also established the link between acquired antibody mediated humoral immunity (HI) with CMI against VVC, suggesting the existence of protective antibodies but at lower concentrations with no appreciable protection (141–143). A common misbelieve regarding IL-17 is that, it is only produced by adaptive Th17 cells and thus function mainly in the adaptive immunity. However, an array of innate immune cells called “innate Type 17” cells also produces IL-17. These cells include innate lymphoid cell type 3 (ILC3), natural killer T (NKT) cells, γδT cells, and TCRβ^+^ “natural” Th17 cells (nTh17) (144–146). Furthermore, contrasting data regarding neutrophils as a source of IL-17 has also been documented (147, 148). However, as aforementioned, CARD9, an adapter that mediates Dectin-1 signaling, is essential for adaptive IL-17 response but this adaptor was not shown to be involved in innate IL-17 responses (126). Recently, a study reported Candidalysin mediated innate IL-17 response in murine model of oral candidiasis (149). However, the role of IL-17 production by innate type 17 cells in VVC is largely uncharted. Overall, these studies highlight the role of innate immunity that work in concurrence with vaginal epithelial cells against VVC, while, the role of adaptive immunity is still elusive (Figure 2).

### Immunity in TV

The innate immunity against *T. vaginalis* involves PRRs stimulation, phagocytes recruitment in vagina and complement activation (150–154). The first barrier of innate immunity encountered by pathogens is vaginal epithelial cells, that lead to immune response generation by TLRs (TLR2, TLR4, and TLR9) expression via p38 MAPK signaling pathway, consequently leading to IL-8 and TNF-α release from vaginal epithelium (155, 156). However, ligands of *T. vaginalis* that bind to these TLRs have not been identified till date. Alternatively, independent of these TLRs expression, the lipophosphoglycan (LPG), a major component of *T. vaginalis* membrane, also induces inflammatory response by release of pro-inflammatory cytokines after contacting human vaginal epithelial cells (157). In addition, galectin-1 and galectin-3 expressed by vaginal epithelial cells were reported as receptors for *T. vaginalis* LPG (158, 159). Galectin-3 was accounted for pro-inflammatory cytokines (IL-8 and MIP-3α) release whereas galectin-1 was shown to play immunosuppressive role that might help in parasites evasion. Both, IL-8 and MIP-3α cytokines show chemotactic activity, promote migration of immune cells particularly neutrophils and other phagocytes across the endothelium, while, MIP-3α also induces dendritic cell maturation (146). Moreover, *T. vaginalis* also releases leukotriene B4 (LTB4), an endogenous lipid, which leads to inflammation in women infected with *T. vaginalis* due to its leukocytes chemotactic activity (160, 161). This lipid mediator induces the release of IL-8, ROS, and AMPs including β-defensin-3 and cathelicidin (LL-37) through binding to its receptors BLT1 and BLT2 on immune cells (162). The immune cells that are predominantly found in the vaginal secretions of *T. vaginalis* infected patients are neutrophils (163). In response to *T. vaginalis* stimulation, immune cells release pro-inflammatory cytokines including IL-1β, IL-6, IL-8, and TNF-α, leading to neutrophils recruitment, explaining its predominant accumulation for mediating initial inflammatory response following TV (161, 164, 165). These neutrophils achieve *T. vaginalis* killing by phagocytosis and by novel mechanism i.e., taking *T. vaginalis* “bites” prior to parasite death, using trogocytosis (166). Immune-fluorescence analysis of the *T. vaginalis* revealed binding of MBL to its surface carbohydrates i.e., unmodified N-glycans (153). MBL binding to *T. vaginalis* have shown to cause self-aggregation of parasites, lowering their motility and division rate (153). *T. vaginalis* also leads to the activation of complement system, encouraging killing through neutrophil-mediated endocytosis (150, 151, 153). Furthermore, SLPI, an AMP, has also shown to be associated with TV (167).

**Figure 3:**
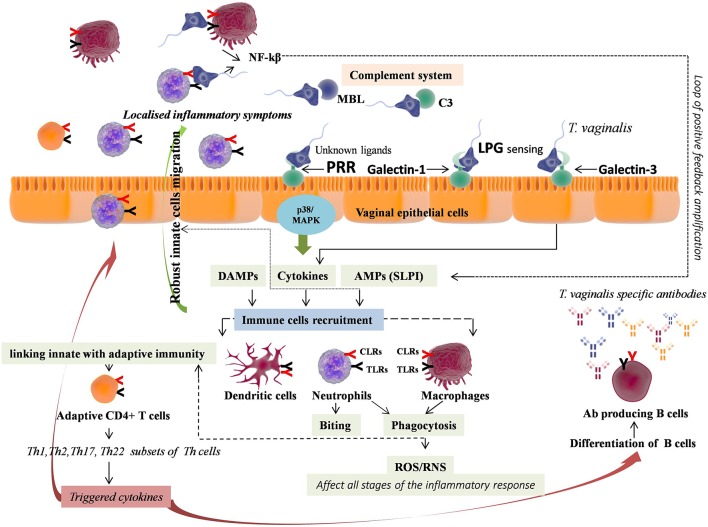
Immunopathology of Trichomoniasis (TV). *T. vaginalis* through unknown ligand lead to immune response generation by pattern recognition receptors (PRRs including TLR2, TLR4, and TLR9) via p38 MAPK signaling pathway, consequently leading to IL-8 and TNF-α release from vaginal epithelium. Independent of this, Galectin-1 and Galectin-3 expressed by vaginal epithelial cells recognize *T. vaginalis* LPG, wherein Galectin-3 accounts for pro-inflammatory cytokines (IL-8 and MIP-3α) release. The pro-inflammatory cytokines promote recruitment and migration of immune cells, with predominant accumulation of neutrophils. The neutrophils achieve *T. vaginalis* killing either by phagocytosis or by novel mechanism *i.e*. taking *T. vaginalis “*bites” prior to parasite death, using trogocytosis. *T. vaginalis* also leads to the activation of complement system, encouraging killing through neutrophil-mediated phagocytosis. Phagocytosis involves reactive oxygen species (ROS) production that further regulates all stages of inflammation. The phagocytised pathogenic components activate NLRP3 inflammasome, which further link innate with adaptive immunity, promoting adaptive CD4^+^ T cells response. The different subsets of Th cells trigger cytokines that collectively lead to the activation and migration of effectors cells promoting extreme vaginal inflammation, along with differentiation of B cells into *T. vaginalis-*specific antibody producing plasma B cells.

Moreover, TV was found to induce CD4^+^ T cells penetration in vaginal tissues indicating the role of T-cell mediated adaptive immunity in defense against TV (168). In support of this, a study reported the involvement of Th1 triggered cytokines (IL-2 and IFN-γ) in maintaining low burden of *T. vaginalis* infection (169). Similarly, the elevated levels of Th17 triggered IL-17 and Th22 triggered IL-22 were found in women with Trichomoniasis (170). Furthermore, elevated expression of cytokines including IL-6 and TNF-α, that induces the differentiation of Th22 cells, were reported in *T. vaginalis* activated macrophages (165). Both, IL-22 and IL-17 share common functional aspects and were shown to collaboratively induce and up-regulate production of an AMP named cathelicidin (LL-37) (170–172). All these cytokines produced by different subsets of Th cells collectively lead to the activation and migration of effectors cells including neutrophils, macrophages, cytotoxic T lymphocytes, natural killer cells, along with differentiation of B cells into antibody producing plasma B cells (173). However, the exact role of these different subtypes of Th cells in TV is still remains to be elucidated. *T. vaginalis* infection also leads to the induction of high concentration of *T. vaginalis* specific IgG, IgM, IgA, and IgG subclass antibodies, along with induction of low concentration of IgE antibodies, in vaginal secretions and serum of *T. vaginalis*-infected subjects (152, 154, 174–177). However, the protective role of these antibodies during *T. vaginalis* infection remains obscure due to their short-lived effects caused by degradation of these antibodies by *T. vaginalis* secreted cysteine proteases (178, 179). Moreover, recently a study has reported direct association between the effective metronidazole based therapy of TV with diminution of specific anti-*T. vaginalis* IgG antibody in serum (177). Thus, just like other VVIs both innate and adaptive immune responses are activated during *T. vaginalis* infection (Figure 3). However, the role of adaptive immunity is still not clear, though elucidated better than VVC and BV.

## Host Defense Against RVVI Leads to Oxidative Stress

A wide range of substances, known as reactive oxidants, consisting of free radicals and other non-radical oxygen derivatives, are constantly generated as an essential part of metabolism. These reactive oxidants are further neutralized by an array of protective antioxidant mechanisms occurring in human system and thus maintains redox homeostasis (180). An imbalance between oxidants and antioxidants, due to an excess oxidants production, leads to oxidative stress that disrupts redox homeostasis (180). From the past decade, evidences have emerged that suggested, reactive oxidants also generate as an integral part of defense mechanism, leading to the state of oxidative stress with significant biological consequences and thus contributing to the pathophysiology of diseases (181). These reactive oxidants are divided in to two main types including reactive oxygen species (ROS) and the reactive nitrogen species (RNS) (182). The former includes primary ROS namely superoxide anion radical (${\text{O}}_{2}^{-}$) and secondary ROS including hydrogen peroxide (H_2_O_2_) and the hydroxyl radical (OH^.^) generated by the action of enzyme nicotinamide adenine dinucleotide phosphate (NADPH) oxidase (NOX) that exists in different isoforms (183). The latter includes nitrogen oxide free radical (NO^.^), the parent molecule of all RNS, generated by the action of enzyme NO synthase (NOS) that catalyse the conversion of l-arginine to l-citrulline in a five-electron oxidative reaction. The NOS exist in three different isoforms that includes type I-neuronal NOS (nNOS), type II-inducible NOS (iNOS), and type III-endothelial NOS (eNOS). In which iNOS is expressed only upon cell stimulation by pro-inflammatory cytokines, by pathogen and pathogen associated molecules (as given below) while nNOS and eNOS express constitutively (184). These reactive oxidants affect all the stages of the inflammatory immune response, starting from the release of DAMPs, their sensing by PRRs, activation of signaling pathways, release of immune mediators, and initiation of the innate and adaptive cellular responses to the ultimate killing of pathogens by effectors phagocytic cells as validated by different studies mentioned above and below.

Oxidative stress plays an important role in release of DAMPs such as HMGB1 in external surroundings (185–187). In turn, HMGB1 itself leads to ROS and RNS generation by stimulating cellular responses and up-regulating genes encoding iNOS via TLR4 activation (188, 189). Moreover, these oxidants also promote cells surface expression of PRRs (190, 191). In turn, activated TLR and CLR-dependent pro-inflammatory signaling, is coupled with ROS generation and up-regulated NOXs and iNOS expression (130, 185, 192, 193). Thus, TLRs and CLRs activation results into both oxidative and nitroxidative stress. These oxidants further leads to TLR engagement and activation resulting in a round of magnification, designated as the “TLR-radical cycle,” which ultimately causes chronic inflammatory response (193). Moreover, the responses generated by PRRs activation are mainly communicated through activation of NF-κB, a redox sensitive transcription factor, which is also activated by ROS. In turn, NF-κB controls the expression of genes involved in innate immunity that leads to ROS generation (194). In addition, ROS generation have also been implicated for NLRP3 activation that generate adaptive immune responses through inflammasome and also leads to further ROS generation (195, 196).

Overall, these studies suggest that both oxidative stress and inflammation stimulate each other and thus create a nasty cycle that leads to the amplification and dissemination of inflammatory response and respiratory/oxidative burst (the production of ROS). The latter is a crucial reaction that occurs in activated inflammatory cells especially neutrophils and macrophages to degrade and kill internalized pathogen by phagocytosis. That is why oxidative burst has long been recognized as a typical consequence of immune cell stimulation coupled with both chronic and acute states of inflammation (182, 197). All these inflammatory stages that lead to ROS generation are shown to be actively involved in RVVI, discussed above, suggesting the major role of oxidative stress in pathophysiology of RVVI and its contribution in vaginal immunity as an integral part of defense mechanism.

Moreover, studies have documented the ROS production by neutrophils in response to pathogenic bacteria, *C. albicans* and *T. vaginalis* infection (43, 130, 163, 198–200). Studies also documented the release of high levels of ROS by neutrophils in response to *Candida* species such as *C. galbrata* and *C. dubliniensis* (201, 202). The concentration of ROS in the vaginal discharge of BV patients was found to be significantly higher than that from healthy women (203). *Candida* species isolated from VVC patients induce ROS production in neutrophils (204). Reduced vaginal concentrations of NO metabolites were shown to increase susceptibility to recurrent episodes of VVC (205). Likewise, reduced serum concentration of ROS has been shown to increase susceptibility to RVVI (206). Other than neutrophils, macrophages were also shown to release cytotoxic NO products against *T. vaginalis* with increased iNOS expression (165, 207). In addition, high levels of cytotoxic NO products and increased iNOS expression was found in WBCs and vaginal lavages of *T. vaginalis* infected asymptomatic women relative to symptomatic women (208, 209). Thus, high ROS production plays an important role in maintaining low burden of infections, as observed in asymptomatic women depicting a strong relationship between oxidative stress and vaginal inflammation caused by RVVI.

## Hijacking or Exploitation of Host Immune Responses by RVVI Pathogens

As discussed above, inflammation signifies an essential immune mechanism which is meant to eliminate pathogens and repair the damage caused by deleterious stimuli. However, there are conditions in which such refurbishment may not occur effectively, resulting in constant pushy cellular stress, disseminating, and magnifying the inflammatory response. In these situations, the process becomes defective, leading considerable variations in tissue functions, with persistent and systemic derangements of homeostasis (182, 210). These conditions usually occur when pathogen becomes capable of evading and subverting host immune responses, creating a niche that allows its replication and leading to continuous stimulation and thus amplification of inflammatory immune responses. Generally, the evasion mechanisms counteract different events in the entire RVVI pathogenesis, but this review will focus on the mechanisms by which pathogens subvert or manipulate the host immune responses, hijack it and use it for its own advantage.

In BV, the biota related to BV was found to inhibit the release of secondary pro-inflammatory cytokine i.e., IL-8 (69). As IL-8 endorses neutrophils migration, its absence does not allow neutrophils to enter the vagina because of which local inflammation does not occur, allowing pathogen survival. Also BVAB were shown to release a combination of short chain fatty acids, including butrate and succinate, which modulate the host immune responses by negatively affecting neutrophils and monocytes migration in vagina and their endocytic activity (211). Moreover, lavages from women with BV as well as *G. vaginalis* separately found to reduce internalization ability of dendritic cells (DC) (91, 92). Additonally, BVAB has been shown to mask itself from host's immune responses by incorporating host sialic acid produced as a result of chopping of vaginal mucosal layer by bacteria (212–214).

Similarly in VVC, *C. albicans* was found to down-regulate TLR4 expression on epithelial cells, thus restraining TLR4 mediated stimulation of immune responses and increasing *C. albicans* infection (101). *Candida* species has the ability to shield its β-glucan (the popular ligand of Dectin-1) with the help of its cell wall components, thus preventing its recognition by Dectin-1 and inhibiting Dectin-1 mediated immune responses (215). As mentioned above, *C. albicans* leads to the activation of complement (C) system and thus generate host immune responses against pathogens. However, the pathogen employs different strategies to evade the classical and alternative pathways of C system. The first strategy includes degradation of host complement components including C3b, C4b, and C5 (216). Second involves surface acquisition of host complement inhibitors or regulators involving plasminogen-binding surface protein, factor H, C4b-binding protein (C4BP), and FHL-1. These surface attached inhibitors sustain their regulatory property and inactivate respective host C proteins (217–219). The third strategy includes direct and indirect C inhibition by self secreting inhibitory protein including pH-regulated Ag 1 (Pra1). This protein either directly blocks the activation and conversion of C3 or indirectly inhibits C system by binding to the host C inhibitor proteins (factor H and C4BP) (220–222). *Candida* also inhibits the terminal complement complex (TCC) formation by secreting Saps proteins (216).

Alternatively, *C. albicans* was shown to inhibit phagolysosomes formation, which is an important step in the process of killing of a pathogen (223). Moreover, pyroptosis is a host antimicrobial response mechanism that involves death of host cells infected with intracellular pathogens. This mechanism has been shown to be hijacked by highly virulent *Candida* strain, for escaping the host immune cells, particularly macrophages, thus mediating its own survival and host cell killing (122, 123, 224, 225). As afore-discussed oxidative stress plays a fundamental role in host defense against RVVI. However, *Candida* has strategies to neutralize and evade the oxidative stress. *C. albicans* expresses superoxide dismutase (SODs) and other antioxidant enzymes on the cell surface. These extracellular SODs also have vital roles in the detoxification of superoxide radicals generated by phagocytes and hence prevent massive ROS accumulation (226, 227). In addition *C. albicans* catalase and vacuole (fungal organelle) formation has been suggested to counteract oxidative stress (228–230). Moreover, *C. albicans* also exploit host cytokine production for its own benefit by inhibiting host IL-12, IL-17, and IFNγ production (231–234). These cytokines are critical for host innate and adaptive immunity as studied above.

Similarly, *T. vaginalis* uses DCs and macrophages for its own advantage by modulating their immune responses leading to reduced synthesis of IL-12 and increase synthesis of IL-10 and TGF-β (235, 236). Furthermore, NF-κB was also shown to be inhibited by *T. vaginalis*, further suppressing the expression of pro-inflammatory genes including IL-12, suggesting the modulation of the host cytokines milieu as an effective immune evasion strategy followed by *T. vaginalis* (235). Other than this, cysteine proteases secreated by *T. vaginalis* helps in evasion of host's immune responses by degrading its various components that includes subclasses of host antibodies (IgG and IgA), C3 opsonin and secretary leukocyte protease inhibitor (SLPI), an antimicrobial peptide (178, 179, 237–239). The cysteine proteases also contribute to cytotoxicity particularly against B cells (240, 241). Killing of B cells and phagocytosis of human peripheral blood mononuclear cells (PBMCs) through contact dependent manner by *T. vaginalis* have also been reported (242, 243). This parasite neutralize specific host antibodies by secreting numerous immunogenic soluble antigens, thereby evading host immune responses (244–246). Additionally, *T. vaginalis* has been suggested to incorporate host serum proteins in its surface, masking itself from host's immune responses (247). Taking this into consideration, recently it was proposed that *T. vaginalis* acquire CD59 from host cells, e.g., red blood cells (RBCs), thereby evading itself from host complement mediated killing (248).

As mentioned above, galectin-1 and galectin-3 receptors expressed on vaginal epithelial cells surface, bind to LPG core of *T. vaginalis* (158, 159). The binding of *T. vaginalis* to galectin-1 plays an immunosuppressive role by inhibiting the releases of IL-8, MIP-3α, and RANTES, the chemokines that connect innate and adaptive immunity and facilitate the recruitment of phagocytes, an another important evasion strategy by the pathogen (159). This suggests that in response to *T. vaginalis* infection, the two molecules of same family play contrasting role. However, in general, galectin-1 can also play immuno-stimulatory role while galectin-3 is also capable of down-regulating the inflammation (249, 250). The possible explanation behind this inconsistency could be recognition of self ligands on host cell surface by galectins, the reason behind why galectins are still not strictly considered as PRRs, because PRRs recognize only highly conserved pathogen associated structure that are not present in host (251). This obvious contradiction divulges our incomplete understanding regarding the structural and biophysical features of ligand binding preferences displayed by galectins and genuine variety in identification of the host galectin range (252). However, the details of this topic are outside the scope of this review. Moreover, *T. vaginalis* can leads to apoptosis of neutrophils and macrophages by reducing expression of the anti-apoptotic proteins and by activating caspase-3, which is a pro-apoptotic marker (253, 254). Other than this, like many other parasites, *T. vaginalis* also releases extracellular vesicles, for instance exosomes, which can also modulate the host immune responses by diminishing vaginal IL-17 concentration and up-regulating IL-10 expression in macrophages (255). Besides this, the surface immunogens of *T. vaginalis*, involving P230 and P270 go through conformational changes that prevent the epitope accessibility for binding of host antibodies that allows parasite to evade host humoral immune responses (256, 257). Thus, understanding the molecular mechanisms employed by pathogens to exploit the host immune response for its own benefits is of crucial importance for the management of these devastating infections.

## Immunopathogenesis of RVVI: Proposed Theory

Vaginal innate immunity is the first line of defense system that responds to the pathogens and activates the adaptive immunity. In turn, activation of adaptive immune responses also support the function of innate immunity consequently forming a loop of positive feedback, leading to amplified inflammatory responses and augmented effectors functions for the ultimate killing of the pathogens. This emphasize on the pivotal role of innate immunity, whose impairment can leads to adaptive immune dysfunction and increased susceptibility to infections. Women inspite of having disturbances in vaginal milieu represent different clinical outcomes. Asymptomatic cases of RVVI is due to proper functioning of both innate and adaptive immune system, that have successfully coped up with the infection in spite of different evasion strategies followed by the pathogens and thus asymptomatic cases should be considered as healthy individuals. While symptomatic cases are characterized by the impairment in the communication part of the innate immunity that signal for the activation of host adaptive immune responses thus, breaking the loop of feedback amplification. This increases the chances of hijacking and evasion of the immune system by the pathogen, which creates a niche for the pathogen replication, leading to continuous stimulation and violent innate immune responses. Thus, symptoms that define the infection are due to robust inflammatory immune responses and high vaginal pathogen burden—a fine interplay that determines the clinical outcome of infection. However, the presence of symptomatic RVVI is appeared to be more dependent on host factors rather than on pathogens itself. Therefore, treatment should depend upon recognition of the impaired unit of innate immunity that do not allow adaptive immunity to responds and thus increasing susceptibility to vaginal infections. These most probable impaired innate immune units could be PRRs that signal for activation of adaptive immune responses. The genetic variations in these PRRs have been shown to play an important role in how a woman responds to a particular microbial challenge, as evidenced by several genetic disease association studies given below. Thus, looking at the women itself provided the answer to the major query suggesting that symptomatic/asymptomatic cases of RVVI are due to differences in women's immunity, conferred partially or wholly by genetic variations.

## Genetic Susceptibility: Host Genotype Modulates Immune Responses and Vulnerability to RVVI

Genetic variations, particularly single nucleotide polymorphisms (SNPs), in genes coding various components of immune system have evidently been shown to modulate innate and acquired antimicrobial immune responses, both qualitatively and quantitatively. These SNPs affect gene expression as well as function and hence modulates individual's susceptibility to acquire diseases including RVVI, as evidence by several studies (Table 1). SNPs in genes coding cytokines, enzymes, growth factor, PRRs, and signaling adaptors were found to be associated with RVVI susceptibility. A study have shown association of dull IL-1β response against pathogens in allele 2 IL1RN^*^2 carriers resulted due to polymorphism in intron 2 (IL1RN) of interleukin-1 receptor antagonist gene (*IL-1Ra*), widely known to modulates the pro-inflammatory action of IL-1 gene (258). Similarly, immunomodulatory effect of *IL1*β polymorphisms was shown to be associated with susceptibility of acquiring BV (259, 260). Another study showed increased susceptibility to RVVC due to reduced levels of vaginal anticandidal factors in *IL-4* polymorphism homozygotes carriers (205). Also, polymorphisms in *IL-6* were associated with reduced cytokine responses, conferring increased risk to BV and premature deliveries (260, 261). However, *IL-8* polymorphism was shown to be associated with increased cytokine responses and decreases BV risk (260). Presence of polymorphisms in tumor necrosis factor-α (*TNF-*α) in BV women were associated with increased vaginal TNF-α levels and preterm deliveries (262, 263). Another study showed association of increased BV risk as well as pre-term delivery with polymorphisms in protein kinase C alpha (*PRKCA*) and fms-like tyrosine kinase 1 (*FLT1*) genes involved in the regulation of inflammatory responses (261). Corticotropin-releasing hormone (CRH) is involved in stress and regulation of inflammatory immune responses. Polymorphisms in genes coding for Corticotropin-releasing hormone binding protein (*CRH-BP*), Corticotropin-releasing hormone (*CRH*) and corticotropin-releasing hormone receptor 2 (*CRH-R2*) were found to be associated with BV (264). Functional polymorphism at position 677 in gene coding for methylene tetrahydrofolate reductase (*MTHFR*), a rate limiting enzyme in methyl cycle, was shown to alter MTHFR activity and DNA methylation in human placenta, which further increases 3.5-fold risk of premature rupture of membranes in BV positive women (265).

**Table 1:** Human genes conferring susceptibility to RVVI.

***Genes***	**SNP(s)^¥^**	**Associated phenotype observed**	**RVVI**	**References**
*IL-1ra*	IL1RN*2	Reduced IL-1β response	Increased colonization of anaerobic Gram-negative rods, *Mycoplasma*, and *Peptostreptococci* and Decreased *Lactobacilli* colonization	(258)
*IL-1β*	−511 and +3954	–	Increased risk for BV	(259)
	+3954	Increased cytokine response	Decreased risk for BV	(260)
*IL-4*	rs2243250 (−589T/C)	Increased vaginal IL-4, reduced NO and MBL levels	Increased risk for RVVC	(205)
*IL-6*	−174	Reduced cytokine response	Increased risk for BV	(260)
	rs1800795	–	BV and High spontaneous preterm delivery	(261)
*IL-8*	−845	Increased cytokine response	Decreased risk for BV	(260)
*TNF-a*	TNF-2 (−308)	–	BV and Increased risk of spontaneous preterm birth	(262)
	−308G>A	Elevated levels of vaginal TNF-α	BV	(263)
*PRKCA*	rs1003599, rs10491202, rs11658528, rs16960112, rs17762314, and rs1990503	–	BV and High spontaneous preterm delivery	(261)
*FLT1*	rs748252	–		
*CRH-BP*	+17487	–	BV	(264)
*CRH*	+ 3362 and −1667	–	Increased risk for BV	
*CRH-R2*	+8288,+ 5253, and + 4853	–	BV	
*MTHFR*	C677T	Altered MTHFR enzyme activity, Affect DNA methylation in the human placenta	BV and increased risk of premature rupture of fetal membranes	(265)
*TLR2*	rs1898830,	–	3 fold increased rate of BV/intermediate flora	(266)
	rs1898830, rs11938228, rs3804099	–	Increased colonization of endometrial anaerobic gram-negative rods. anaerobic non-pigmented Gram-negative rods, anaerobic Gram-positive cocci	
	rs3804099	–	Decreased risk of BV	(267)
	rs1898830	–	Increased risk of BV	(268)
	rs5743704 (P631H)	Deleterious effects on protein function; reduces production of IL-17 and IFNγ	3-fold increased risk of RVVC	(269)
*TLR4*	896 A > G	Reduced vaginal IL-1β and IL-1ra levels	>10-fold increased colonization of *Gardnerella vaginalis* and anaerobic Gram-negative rods, *Prevotella, Bacteroides, and Porphyromonas*.	(71)
	rs4986790	–	Increased risk of BV	(268)
*TLR7*	rs5743737 and rs1634323	–	Decreased risk of BV	(267)
	rs179012	–	Increased risk of BV	
*TLR9*	rs187084	–	Increased risk of BV	(268)
*CIAS1*	Tandem repeat in intron 4	Impaired NLRP3 expression and IL-1β production	RVVC	(270)
*MBL2*	Codon 54 (rs1800450)	Low MBL levels in cervico-vaginal fluids	Increased risk of RVVC	(107, 108, 271, 272)
		–	Increased risk of both RBV and RVVC	(273)
	Y/X(rs7096206)	Low sMBL levels	Increased risk of RVVI either it is BV, VVC or MI	(55)
	rs10824792	Low sMBL levels	Increased risk of RVVI either it is BV, VVC or MI	(274)
	rs7084554 and rs36014597	Low sMBL levels	Increased risk of RVVI either it is BV, VVC or MI	(275)
*CLEC7A*	rs16910526 (Y238X)	Poor dectin-1 expression, defective ligand binding, defective immune responses	Increased risk of RVVC	(129)
	rs3901533	High sdectin-1 levels	Decreased risk of RVVI either it is BV, VVC, or MI	(57)
*CARD9*	Q295X	Decreased proportion of Th17 cells	RVVC	(276)

¥ *represented either by rs number, position or by possible allele, See text for required abbreviations*.

As discussed above, PRRs are the important dictatorial components of immune system. Polymorphisms in gene encoding PRRs including TLRs (*TLR2, TLR4, TLR7*, and *TLR9*), NLR (*CIAS1*), and CLRs (*MBL2* and *CLEC7A*) have been shown to modulate immune responses and susceptibility to RVVI. Studies have shown association of *TLR2* polymorphisms with 3-fold increased risk of acquiring BV and increased colonization of BVAB (266, 268). In consonance, a non-synonymous SNP (nsSNP) in *TLR2* was linked with defective protein function, which subsequently reduced the production of pro-inflamatory cytokines and predisposition to RVVC (269). Similarly, polymorphisms in *TLR4, TLR7*, and *TLR9* were shown to be associated with increased risk of BV and >10-fold increased colonization of BVAB (71, 267, 268). In contrast, a study has shown decreased risk of acquiring BV with *TLR2* and *TLR7* Polymorphisms (267). Additionally, polymorphism in *NLRP3* gene, also known as cold-induced auto-inflammatory syndrome 1 (*CIAS1*) gene, which code for the inflammasome component NLRP3, has been shown to cause impaired NLRP3 expression and IL-1β production that subsequently predisposes women to RVVC (270). Moreover, polymorphisms in genes involving *MBL2* and *CLEC7A* have been shown to modulate their encoded CLRs expression and activity resulting in defective immune responses and altered susceptibility to RVVI (55, 57, 107, 108, 129, 271–273). Studies have shown an association of *MBL2* codon 54 polymorphisms with increased RVVC risk (107, 108, 271, 272). Another study has found an association of codon 54 polymorphism with increased risk of both RVVC and RBV (273). Moreover, Y/X promoter polymorphism of *MBL2* was found to predispose women to RVVI either it is BV, VVC, or MI in North Indian population (55). A study identified and explained a *CLEC7A* nsSNP i.e., Y238X (rs16910526) in antifungal defenses in onychomycosis and RVVC (129). The study showed poor expression of mutated form of Dectin-1, with defective β-glucan binding, defective Th17 responses, and defective production of cytokines including IL-6, TNF, and IL-17, alluring the cause of VVC in these patients. Moreover, Y238X variant was depicted to show gene-dose effects i.e., onset of disease at early age of 10–12 years was observed in homozygous variant daughter relative to late onset at age of 40 and 55 years in heterozygous mother and father, respectively (129). Recently, another study has reported that G allele of *CLEC7A* rs3901533 intronic variant and its homozygous carriers significantly lower the risk of developing RVVI and its types i.e., BV, VVC, or MI in North Indian population (57). Furthermore, a variation (Q295X) in *CARD9*, coding for adaptor (CARD9) that mediates Dectin-1 signaling, was shown to impair Dectin-1 signaling, resulting in decreased numbers of interleukin-17 producing effector Th17 cells and increased risk of RVVC (276). In addition, precision in diagnosing RVVI and subject assortment lead to variations between studies that limit the simplification of the reported findings to other populations.

## Research Directions

Though, understanding regarding immunopathology of recurrent vulvovaginal infections has broadened recently, still there are many key questions that are needed to be addressed.

First, unlike VVC and TV, studies have provided no relevant evidence for neutrophils elevation in BV, though evidences regarding high levels of bio-markers including cathelicidin that induces neutrophils migration in BV is present (88, 89).

Second, candidalysin has recently been proposed as a key hypha-associated virulence factor responsible for immunopathogenesis of VVC (96). However, the receptors employed by epithelial cells to sense candidalysin activity are still pending to be eludicated.

Third, pyroptosis is employed as one of the evasion strategies by virulent *Candida* for escaping macrophages (122, 123). However, the fungal triggers activating the inflammasome mediated pyroptosis are still not known.

Fourth, both *in vitro* and *in vivo* studies in VVC have shown that Dectin-1-Syk-CARD9 signaling, couple innate and adaptive immunity independently of TLR signals and induce differentiation of adaptive Th-17 and Th-1 cells (125, 126). However, no such role of Dectin-1 has been reported in BV and TV till date. Though, its role in defense and recognition of these pathogens has been revealed (277, 278). However, these pathogens do not possess β-glucans, suggesting the possibility of other ligands of Dectin-1 that are still not identified.

Fifth, an array of innate immune cells called “innate Type 17” cells have also been shown to produces IL-17 (144–146). Recently, a study reported candidalysin mediated innate IL-17 response in murine model of oral candidiasis (149). However, the role of IL-17 production by innate type 17 cells in BV, VVC, and TV is largely uncharted.

Sixth, the first barrier of innate immunity encountered by pathogens is vaginal epithelial cells that lead to immune response generation by TLRs (155, 156). However, ligands of *T. vaginalis* that bind to these TLRs have not been identified till date.

Seventh, the odds of hijacking increases, due to impaired immune responses, the net magnitude of which is the result of numerous genetic variations, present in multiple host genes, detailed in this review. However, so far, the functional consequences of genetic variations of only two genes i.e., *MBL2* and *CLEC7A* have been reported, while the role of other associated genetic polymorphisms are still pending to be elucidated.

Finally, valuable information relative to the role of adaptive immunity in RVVI is still not clear, though elucidated better in TV than VVC and BV.

## Treatment Strategies

Advancements in diagnostic tools as well as drugs, targeting the pathogens, provide temporarily relief, as the infection re-occurs because disturbance in host genetic system is still persisting that must be rectified in order to restore host homeostasis. The afore-highlighted causal factors, that modulate propensity to RVVI in women, may further be used to develop efficient diagnosis and treatment strategies of this enigmatic disease. As for instance,

### MBL Replacement Therapy

Plasma-derived MBL replacement has become a safe and efficacious therapeutic option in diseases associated with low MBL levels (279–281). Therefore, the emerging MBL substitution therapy could possibly be the future treatment strategy for RVVI (282).

### Adoptive T-Cell Therapy

The use of CD8^+^, CD4^+^, and γδ T cells for the treatment of infections dieases and cancer has been reported to safe and efficacious (283). Therefore, the emerging MBL substitution adoptive T-Cell therapy could possibly be the future treatment strategy for RVVI.

### Antibody-Based Therapy

Presence of many licensed monoclonal antibodies for the treatment of infectious diseases including HIV (284) outlines the prospects of this therapy for RVVI.

## Conclusions

**Figure 4:**
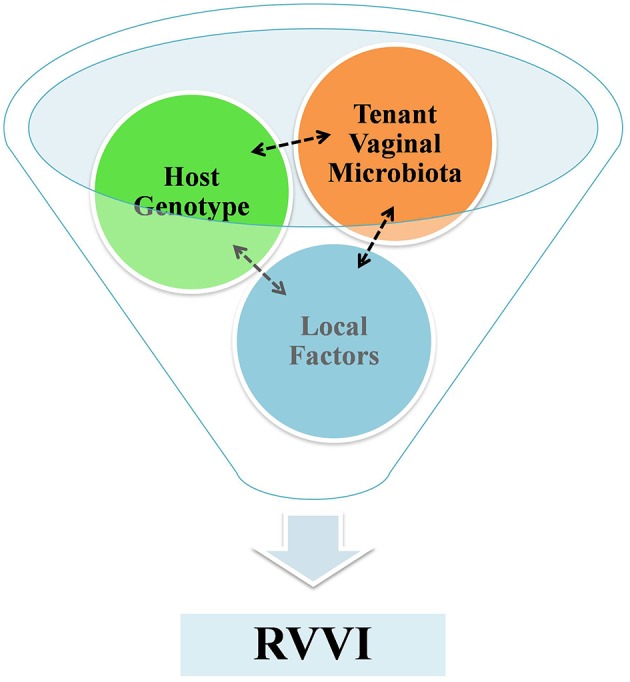
A funnel representing a fine interplay between host VMB, host genotype and local factors that culminates to symptomatic RVVI. The tenant VMB controls host gene expression and in succession the tenant VMB is shaped by host genotype, while exposures of both local systemic and environmental factors influence VMB and host genome.

The methodical scrutiny of literature indicated RVVI as a multifarious disease, requiring a “perfect storm” to start infection. This “perfect storm” is a result of fine interplay between host VMB, host genotype and other local risk factors that culminate into symptomatic infection (Figure 4). However, the presence of symptomatic RVVI is appeared to be more dependent on host factors rather than on pathogens itself. Thus, by underlining the role of the host immune responses in disease etiology, modern research has clarified a major hypothesis shift in the philosophy of RVVI pathogenesis. Future research in explication of highlighted critical questions may provide complete understanding of immunopathological mechanisms of RVVI. Future research in explication of the highlighted causal factors, that modulate propensity to RVVI in women, may reveal new biologicals for preventing and treating RVVI.

## Author Contributions

NK collected the literature and wrote the manuscript. JS and MK designed and critically checked the manuscript.

### Conflict of Interest Statement

The authors declare that the research was conducted in the absence of any commercial or financial relationships that could be construed as a potential conflict of interest.

## References

[1] Koenig M Jejeebhoy S Singh S Sridhar S Investigating women's s gynaecological morbidity in India: Not just another KAP survey. Reproduc Health Matters. (1998) 6:84–97. 10.1016/S0968-8080(98)90085-4

[2] Mulu W Yimer M Zenebe Y Abera B. Common causes of vaginal infections and antibiotic susceptibility of aerobic bacterial isolates in women of reproductive age attending at Felegehiwot referral Hospital, Ethiopia: a cross sectional study. BMC Women's Health. (2015) 15:42. 10.1186/s12905-015-0197-y 25968607 PMC4438529

[3] Powell AM Nyirjesy P. Recurrent vulvovaginitis. Best Pract Res Clin Obstetr Gynaecol. (2014) 28:967–76. 10.1016/j.bpobgyn.2014.07.006 25220102

[4] Niccolai LM Kopicko JJ Kassie A Petros H Clark RA Kissinger P. Incidence and predictors of reinfection with *Trichomonas vaginalisin* HIV-infected women. Sexually Trans Dis. (2000) 27:284–8. 10.1097/00007435-200005000-00009 10821602

[5] Sobel JD Subramanian C Foxman B Fairfax M Gygax SE. Mixed vaginitis—more than coinfection and with therapeutic implications. Curr Infect Dis Rep. (2013) 15:104–8. 10.1007/s11908-013-0325-5 23354954

[6] Kalia N Singh J Sharma S Kamboj SS Arora H Kaur M Prevalence of vulvovaginal infections and species specific distribution of vulvovaginal candidiasis in married women of north india. Int J Curr Microbiol App Sci. (2015) 4:253–66.

[7] Masha SC Cools P Sanders EJ Vaneechoutte M Crucitti T. *Trichomonas vaginalis* and HIV infection acquisition: a systematic review and meta-analysis. Sex Transm Infect. (2019) 95:36–42. 10.1136/sextrans-2018-053713 30341233 PMC6580735

[8] van Oostrum N De Sutter P Meys J Verstraelen H. Risks associated with bacterial vaginosis in infertility patients: a systematic review and meta-analysis. Hum Reproduc. (2013) 28:1809–15. 10.1093/humrep/det096 23543384

[9] Toth B Würfel W Bohlmann MK Gillessen-Kaesbach G Nawroth F Rogenhofer N. Recurrent miscarriage: diagnostic and therapeutic procedures. guideline of the DGGG (S1-Level, AWMF Registry No. 015/050, December 2013). Geburtshilfe Frauenheilkunde. (2015) 75:1117. 10.1055/s-0035-1558299 26997666 PMC4795844

[10] Vodstrcil LA Twin J Garland SM Fairley CK Hocking JS Law MG. The influence of sexual activity on the vaginal microbiota and *Gardnerella vaginalis* clade diversity in young women. PLoS ONE. (2017) 12:e0171856. 10.1371/journal.pone.0171856 28234976 PMC5325229

[11] Ravel J Gajer P Abdo Z Schneider GM Koenig SS McCulle SL. Vaginal microbiome of reproductive-age women. Proc Natl Acad Sci. (2011) 108(Suppl. 1):4680–7. 10.1073/pnas.1002611107 20534435 PMC3063603

[12] Macklaim JM Fernandes AD Di Bella JM Hammond JA Reid G Gloor GB. Comparative meta-RNA-seq of the vaginal microbiota and differential expression by *Lactobacillus iners* in health and dysbiosis. Microbiome. (2013) 1:12. 10.1186/2049-2618-1-12 24450540 PMC3971606

[13] Petricevic L Domig KJ Nierscher FJ Sandhofer MJ Fidesser M Krondorfer I. Characterisation of the vaginal Lactobacillus microbiota associated with preterm delivery. Sci Rep. (2014) 4:5136. 10.1038/srep05136 24875844 PMC4038809

[14] Falkow S Molecular Koch's postulates applied to microbial pathogenicity. Rev Infect Dis. (1988) 1988:S274–6. 10.1093/cid/10.Supplement_2.S274 3055197

[15] Gonçalves B Ferreira C Alves CT Henriques M Azeredo J Silva S. Vulvovaginal candidiasis: epidemiology, microbiology and risk factors. Crit Rev Microbiol. (2016) 42:905–27. 10.3109/1040841X.2015.1091805 26690853

[16] van de Wijgert JH Borgdorff H Verhelst R Crucitti T Francis S Verstraelen H. The vaginal microbiota: what have we learned after a decade of molecular characterization? PLoS ONE. (2014) 9:e105998. 10.1371/journal.pone.0105998 25148517 PMC4141851

[17] Medzhitov R. Origin and physiological roles of inflammation. Nature. (2008) 454:428. 10.1038/nature07201 18650913

[18] Wira CR Fahey JV Sentman CL Pioli PA Shen L. Innate and adaptive immunity in female genital tract: cellular responses and interactions. Immunol Rev. (2005) 206:306–35. 10.1111/j.0105-2896.2005.00287.x 16048557

[19] Kaushic C Ferreira VH Kafka JK Nazli A. HIV infection in the female genital tract: discrete influence of the local mucosal microenvironment. Am J Reproduc Immunol. (2010) 63:566–75. 10.1111/j.1600-0897.2010.00843.x 20384619

[20] Hickey DK Patel MV Fahey JV Wira CR. Innate and adaptive immunity at mucosal surfaces of the female reproductive tract: stratification and integration of immune protection against the transmission of sexually transmitted infections. J Reproduc Immunol. (2011) 88:185–94. 10.1016/j.jri.2011.01.005 21353708 PMC3094911

[21] Moncla BJ Chappell CA Debo BM Meyn LA. The effects of hormones and vaginal microflora on the glycome of the female genital tract: cervical-vaginal fluid. PLoS ONE. (2016) 11:e0158687. 10.1371/journal.pone.0158687 27437931 PMC4954690

[22] Petrova MI Lievens E Malik S Imholz N Lebeer S. Lactobacillus species as biomarkers and agents that can promote various aspects of vaginal health. Front Physiol. (2015) 6:81. 10.3389/fphys.2015.00081 25859220 PMC4373506

[23] Wira CR Patel MV Ghosh M Mukura L Fahey JV. Innate immunity in the human female reproductive tract: endocrine regulation of endogenous antimicrobial protection against HIV and other sexually transmitted infections. Am J Reproduc Immunol. (2011) 65:196–211. 10.1111/j.1600-0897.2011.00970.x 21294805 PMC3837338

[24] Kurita T. Developmental origin of vaginal epithelium. Differentiation. (2010) 80:99–105. 10.1016/j.diff.2010.06.007 20638775 PMC2943051

[25] Kono H Rock KL. How dying cells alert the immune system to danger. Nat Rev Immunol. (2008) 8:279. 10.1038/nri2215 18340345 PMC2763408

[26] Kumar H Kawai T Akira S. Pathogen recognition by the innate immune system. Int Rev Immunol. (2011) 30:16–34. 10.3109/08830185.2010.529976 21235323

[27] Jang JH Shin HW Lee JM Lee HW Kim EC Park SH. An overview of pathogen recognition receptors for innate immunity in dental pulp. Mediat Inflamm. (2015) 2015:794143. 10.1155/2015/794143 26576076 PMC4630409

[28] Fichorova RN Cronin AO Lien E Anderson DJ Ingalls RR. Response to Neisseria gonorrhoeae by cervicovaginal epithelial cells occurs in the absence of toll-like receptor 4-mediated signaling. J Immunol. (2002) 168:2424–32. 10.4049/jimmunol.168.5.2424 11859134

[29] Fazeli A Bruce C Anumba DO. Characterization of Toll-like receptors in the female reproductive tract in humans. Hum Reproduc. (2005) 20:1372–8. 10.1093/humrep/deh775 15695310

[30] Hart KM Murphy AJ Barrett KT Wira CR Guyre PM Pioli PA. Functional expression of pattern recognition receptors in tissues of the human female reproductive tract. J Reproduc Immunol. (2009) 80:33–40. 10.1016/j.jri.2008.12.004 19406482 PMC2744441

[31] Brown GD. Dectin-1: a signalling non-TLR pattern-recognition receptor. Nat Rev Immunol. (2006) 6:33. 10.1038/nri1745 16341139

[32] Bulla R De Seta F Radillo O Agostinis C Durigutto P Pellis V. Mannose-binding lectin is produced by vaginal epithelial cells and its level in the vaginal fluid is influenced by progesterone. Mol Immunol. (2010) 48:281–6. 10.1016/j.molimm.2010.07.016 20728220

[33] Pioli PA Amiel E Schaefer TM Connolly JE Wira CR Guyre PM. Differential expression of Toll-like receptors 2 and 4 in tissues of the human female reproductive tract. Infect Immun. (2004) 72:5799–806. 10.1128/IAI.72.10.5799-5806.2004 15385480 PMC517561

[34] Hirata T Osuga Y Hirota Y Koga K Yoshino O Harada M. Evidence for the presence of toll-like receptor 4 system in the human endometrium. J Clin Endocrinol Metabol. (2005) 90:548–56. 10.1210/jc.2004-0241 15509642

[35] Schaefer TM Desouza K Fahey JV Beagley KW Wira CR. Toll-like receptor (TLR) expression and TLR-mediated cytokine/chemokine production by human uterine epithelial cells. Immunology. (2004) 112:428–36. 10.1111/j.1365-2567.2004.01898.x 15196211 PMC1782499

[36] Bechinger B Gorr SU. Antimicrobial peptides: mechanisms of action and resistance. J Dental Res. (2017) 96:254–60. 10.1177/0022034516679973 27872334 PMC5298395

[37] Aboud L Ball TB Tjernlund A Burgener A. The role of serpin and cystatin antiproteases in mucosal innate immunity and their defense against HIV. Am J Reproduc Immunol. (2014) 71:12–23. 10.1111/aji.12166 24325760

[38] Wilson SS Wiens ME Smith JG. Antiviral mechanisms of human defensins. J Mol Biol. (2013) 425:4965–80. 10.1016/j.jmb.2013.09.038 24095897 PMC3842434

[39] Yarbrough VL Winkle S Herbst-Kralovetz MM. Antimicrobial peptides in the female reproductive tract: a critical component of the mucosal immune barrier with physiological and clinical implications. Hum Reproduc Update. (2014) 21:353–77. 10.1093/humupd/dmu065 25547201

[40] Pellis V De Seta F Crovella S Bossi F Bulla R Guaschino S. Mannose binding lectin and C3 act as recognition molecules for infectious agents in the vagina. Clin Exp Immunol. (2005) 139:120–6. 10.1111/j.1365-2249.2005.02660.x 15606621 PMC1809267

[41] Volanakis JE (Ed.). Overview of the complement system. In: Frank MM, editor. The Human Complement System in Health and Disease. Informa Health Care. New York, NY: Marcel Dekker (1998). p. 9–32. 10.1201/b14212-3

[42] Givan AL White HD Stern JE Colby E Guyre PM Wira CR. Flow cytometric analysis of leukocytes in the human female reproductive tract: comparison of fallopian tube, uterus, cervix, and vagina. Am J Reproduc Immunol. (1997) 38:350–9. 10.1111/j.1600-0897.1997.tb00311.x 9352027

[43] Nauseef WM. How human neutrophils kill and degrade microbes: an integrated view. Immunol Rev. (2007) 219:88–102. 10.1111/j.1600-065X.2007.00550.x 17850484

[44] Moffett-King A Entrican G Ellis S Hutchinson J Bainbridge D. Natural killer cells and reproduction. TRENDS Immunol. (2002) 23:332–3. 10.1016/S1471-4906(02)02261-5 12103342

[45] Iijima N Thompson JM Iwasaki A. Dendritic cells and macrophages in the genitourinary tract. Mucosal Immunol. (2008) 1:451. 10.1038/mi.2008.57 19079212 PMC2684461

[46] Sallusto F Lanzavecchia A. The instructive role of dendritic cells on T-cell responses. Arthritis Res Ther. (2002) 4:S127. 10.1186/ar567 12110131 PMC3240143

[47] Russell MW Mestecky J. Humoral immune responses to microbial infections in the genital tract. Microbes Infect. (2002) 4:667–77. 10.1016/S1286-4579(02)01585-X 12048036

[48] Wang YY Kannan A Nunn KL Murphy MA Subramani DB Moench T. IgG in cervicovaginal mucus traps HSV and prevents vaginal Herpes infections. Mucosal Immunol. (2014) 7:1036. 10.1038/mi.2013.120 24496316 PMC4122653

[49] Zhou JZ Way SS Chen K Immunology of the uterine and vaginal mucosae. Trends Immunol. (2018) 39:302–14. 10.1016/j.it.2018.01.007 29433961

[50] McKinnon LR Nyanga B Chege D Izulla P Kimani M Huibner S. Characterization of a human cervical CD4+ T cell subset coexpressing multiple markers of HIV susceptibility. J Immunol. (2011) 187:6032–42. 10.4049/jimmunol.1101836 22048765

[51] Figueiredo AS Schumacher A. The T helper type 17/regulatory T cell paradigm in pregnancy. Immunology. (2016) 148:13–21. 10.1111/imm.12595 26855005 PMC4819144

[52] Al-Harthi L Spear GT Hashemi FB Landay A Sha BE Roebuck KA. A human immunodeficiency virus (HIV)-inducing factor from the female genital tract activates HIV-1 gene expression through the κB enhancer. J Infect Dis. (1998) 178:1343–51. 10.1086/314444 9780254

[53] Zariffard MR Novak RM Lurain N Sha BE Graham P Spear GT. Induction of tumor necrosis factor–α secretion and toll-like receptor 2 and 4 mRNA expression by genital mucosal fluids from women with bacterial vaginosis. J Infect Dis. (2005) 191:1913–21. 10.1086/429922 15871126

[54] Anahtar MN Byrne EH Doherty KE Bowman BA Yamamoto HS Soumillon M. Cervicovaginal bacteria are a major modulator of host inflammatory responses in the female genital tract. Immunity. (2015) 42:965–76. 10.1016/j.immuni.2015.04.019 25992865 PMC4461369

[55] Kalia N Singh J Sharma S Arora H Kaur M. Genetic and phenotypic screening of mannose-binding lectin in relation to risk of recurrent vulvovaginal infections in women of North India: a prospective cohort study. Front Microbiol. (2017) 8:75. 10.3389/fmicb.2017.00075 28197138 PMC5281598

[56] Rogers H Williams DW Feng GJ Lewis MA Wei XQ. Role of bacterial lipopolysaccharide in enhancing host immune response to Candida albicans. Clin Dev Immunol. (2013) 2013:320168. 10.1155/2013/320168 23401696 PMC3563236

[57] Kalia N Kaur M Sharma S Singh J. A comprehensive in silico analysis of regulatory SNPs of human CLEC7A gene and its validation as genotypic and phenotypic disease marker in Recurrent Vulvovaginal Infections. Front Cell Infect Microbiol. (2018) 8:65. 10.3389/fcimb.2018.00065 29616193 PMC5869923

[58] Jespers V Kyongo J Joseph S Hardy L Cools P Crucitti T. A longitudinal analysis of the vaginal microbiota and vaginal immune mediators in women from sub-Saharan Africa. Sci Rep. (2017) 7:11974. 10.1038/s41598-017-12198-6 28931859 PMC5607244

[59] Lennard K Dabee S Barnabas SL Havyarimana E Blakney A Jaumdally SZ. Microbial composition predicts genital tract inflammation and persistent bacterial vaginosis in South African adolescent females. Infect Immun. (2018) 86:e00410–17. 10.1128/IAI.00410-17 29038128 PMC5736802

[60] Libby EK Pascal KE Mordechai E Adelson ME Trama JP. Atopobium vaginae triggers an innate immune response in an *in vitro* model of bacterial vaginosis. Microbes Infect. (2008) 10:439–46. 10.1016/j.micinf.2008.01.004 18403235

[61] Eade CR Diaz C Wood MP Anastos K Patterson BK Gupta P. Identification and characterization of bacterial vaginosis-associated pathogens using a comprehensive cervical-vaginal epithelial coculture assay. PLoS ONE. (2012) 7:e50106. 10.1371/journal.pone.0050106 23166828 PMC3499514

[62] Marconi C Donders GG Parada CM Giraldo PC da Silva MG. Do Atopobium vaginae, Megasphaera sp. and Leptotrichia sp change the local innate immune response and sialidase activity in bacterial vaginosis? Sex Transm Infect. (2013) 89:167–73. 10.1136/sextrans-2012-050616 23076402

[63] Doerflinger SY Throop AL Herbst-Kralovetz MM. Bacteria in the vaginal microbiome alter the innate immune response and barrier properties of the human vaginal epithelia in a species-specific manner. J Infect Dis. (2014) 209:1989–99. 10.1093/infdis/jiu004 24403560

[64] Vick EJ Park HS Huff KA Brooks KM Farone AL Farone MB. Gardnerella vaginalis triggers NLRP3 inflammasome recruitment in THP-1 monocytes. J Reproduc Immunol. (2014) 106:67–75. 10.1016/j.jri.2014.08.005 25280956

[65] Imseis HM Greig PC Livengood CH III Shunior E Durda P Erikson M. Characterization of the inflammatory cytokines in the vagina during pregnancy and labor and with bacterial vaginosis. J Soc Gynecol Invest. (1997) 4:90–4. 10.1177/107155769700400208 9101468

[66] Sturm-Ramirez K Gaye-Diallo A Eisen G Mboup S Kanki PJ. High levels of tumor necrosis factor—α and interleukin-1β in bacterial vaginosis may increase susceptibility to human immunodeficiency virus. J Infect Dis. (2000) 182:467–73. 10.1086/315713 10915077

[67] Spandorfer SD Neuer A Giraldo PC Rosenwaks Z Witkin SS. Relationship of abnormal vaginal flora, proinflammatory cytokines and idiopathic infertility in women undergoing IVF. J Reproduc Med. (2001) 46:806–10. 11584481

[68] Cauci S Driussi S Guaschino S Isola M Quadrifoglio F. Correlation of local interleukin-1beta levels with specific IgA response against *Gardnerella vaginalis* cytolysin in women with bacterial vaginosis. Am J Reproduc Immunol. (2002) 47:257–64. 10.1034/j.1600-0897.2002.01096.x 12148539

[69] Cauci S Guaschino S de Aloysio D Driussi S De Santo D Penacchioni P. Interrelationships of interleukin-8 with interleukin-1β and neutrophils in vaginal fluid of healthy and bacterial vaginosis positive women. Mol Hum Reproduc. (2003) 9:53–8. 10.1093/molehr/gag003 12529421

[70] Alvarez-Olmos MI Barousse MM Rajan L Van Der Pol BJ Fortenberry D Orr D. Vaginal lactobacilli in adolescents: presence and relationship to local and systemic immunity, and to bacterial vaginosis. Sexually Trans Dis. (2004) 31:393–400. 10.1097/01.OLQ.0000130454.83883.E9 15215693

[71] Genc MR Vardhana S Delaney ML Onderdonk A Tuomala R Norwitz E. Relationship between a toll-like receptor-4 gene polymorphism, bacterial vaginosis-related flora and vaginal cytokine responses in pregnant women. Eur J Obstetr Gynecol Reproduct Biol. (2004) 116:152–6. 10.1016/j.ejogrb.2004.02.010 15358455

[72] Basso B Giménez F López C IL-1b, IL-6 and IL-8 levels in gyneco-obstetric infections. Infect Dis Obstetr Gynecol. (2005) 13:207–11. 10.1155/2005/358107 PMC1784579 16338780

[73] Marconi C Santos-Greatti MM Parada CM Pontes A Pontes AG Giraldo PC Cervicovaginal levels of proinflammatory cytokines are increased during chlamydial infection in bacterial vaginosis but not in lactobacilli-dominated flora. J Lower Genital Tract Dis. (2014) 18:261–5. 10.1097/LGT.0000000000000003 24633167

[74] Thurman AR Kimble T Herold B Mesquita PM Fichorova RN Dawood HY. Bacterial vaginosis and subclinical markers of genital tract inflammation and mucosal immunity. AIDS Res Hum Retroviruses. (2015) 31:1139–52. 10.1089/aid.2015.0006 26204200 PMC4651020

[75] Alcaide ML Rodriguez VJ Brown MR Pallikkuth S Arheart K Martinez O. High levels of inflammatory cytokines in the reproductive tract of women with BV and engaging in intravaginal douching: a cross-sectional study of participants in the women interagency HIV study. AIDS Res Hum Retroviruses. (2017) 33:309–17. 10.1089/aid.2016.0187 27897054 PMC5372759

[76] Yudin MH Landers DV Meyn L Hillier SL. Clinical and cervical cytokine response to treatment with oral or vaginal metronidazole for bacterial vaginosis during pregnancy: a randomized trial. Obstetrics Gynecol. (2003) 102:527–34. 10.1016/S0029-7844(03)00566-0 12962937

[77] Lopez-Castejon G Brough D. Understanding the mechanism of IL-1β secretion. Cytokine Growth Factor Rev. (2011) 22:189–95. 10.1016/j.cytogfr.2011.10.001 22019906 PMC3714593

[78] Eskan MA Benakanakere MR Rose BG Zhang P Zhao J Stathopoulou P. Interleukin-1β modulates proinflammatory cytokine production in human epithelial cells. Infect Immun. (2008) 76:2080–9. 10.1128/IAI.01428-07 18332211 PMC2346720

[79] Platz-Christensen JJ Mattsby-Baltzer I Thomsen P Wiqvist N Endotoxin and interleukin-1α in the cervical mucus and vaginal fluid of pregnant women with bacterial vaginosis. Am J Obstetrics Gynecol. (1993) 169:1161–6. 10.1016/0002-9378(93)90274-M 8238178

[80] Masson L Arnold KB Little F Mlisana K Lewis DA Mkhize N. Inflammatory cytokine biomarkers to identify women with asymptomatic sexually transmitted infections and bacterial vaginosis who are at high risk of HIV infection. Sex Transm Infect. (2016) 92:186–93. 10.1136/sextrans-2015-052072 26511781 PMC6801014

[81] Balu RB Savitz DA Ananth CV Hartmann KE Miller WC Thorp JM. Bacterial vaginosis and vaginal fluid defensins during pregnancy. Am J Obstetrics Gynecol. (2002) 187:1267–71. 10.1067/mob.2002.126989 12439518

[82] Valore EV Wiley DJ Ganz T. Reversible deficiency of antimicrobial polypeptides in bacterial vaginosis. Infect Immun. (2006) 74:5693–702. 10.1128/IAI.00524-06 16988245 PMC1594936

[83] Orfanelli T Jayaram A Doulaveris G Forney LJ Ledger WJ Witkin SS. Human epididymis protein 4 and secretory leukocyte protease inhibitor in vaginal fluid: relation to vaginal components and bacterial composition. Reproduc Sci. (2014) 21:538–42. 10.1177/1933719113503416 24023032 PMC5933185

[84] Nasioudis D Linhares IM Ledger WJ Witkin SS. Bacterial vaginosis: a critical analysis of current knowledge. BJOG. (2017) 124:61–9. 10.1111/1471-0528.14209 27396541

[85] Imbert M Blondeau R. On the iron requirement of lactobacilli grown in chemically defined medium. Curr Microbiol. (1998) 37:64–6. 10.1007/s002849900339 9625793

[86] Frew L Makieva S McKinlay AT McHugh BJ Doust A Norman JE. Human cathelicidin production by the cervix. PLoS ONE. (2014) 9:e103434. 10.1371/journal.pone.0103434 25089904 PMC4121085

[87] Xhindoli D Pacor S Benincasa M Scocchi M Gennaro R Tossi A. The human cathelicidin LL-37—A pore-forming antibacterial peptide and host-cell modulator. Biochim Biophys Acta. (2016) 1858:546–66. 10.1016/j.bbamem.2015.11.003 26556394

[88] Nijnik A Hancock RE The roles of cathelicidin LL-37 in immune defenses and novel clinical applications. Curr Opin Hematol. (2009) 16:41–7. 10.1097/MOH.0b013e32831ac517 19068548

[89] Pino A Giunta G Randazzo CL Caruso S Caggia C Cianci A. Bacterial biota of women with bacterial vaginosis treated with lactoferrin: an open prospective randomized trial. Microb Ecol Health Dis. (2017) 28:1357417.e1–e11. 10.1080/16512235.2017.1357417 28959181 PMC5614382

[90] Giraldo PC de Carvalho JBJ do Amaral RLG da Silveira Gonçalves AK Eleutério J Guimarães F. Identification of immune cells by flow cytometry in vaginal lavages from women with vulvovaginitis and normal microflora. Am J Reproduc Immunol. (2012) 67:198–205. 10.1111/j.1600-0897.2011.01093.x 22151521

[91] John EPS Martinson J Simoes JA Landay AL Spear GT Dendritic cell activation and maturation induced by mucosal fluid from women with bacterial vaginosis. Clin Immunol. (2007) 125:95–102. 10.1016/j.clim.2007.06.004 17652029 PMC2040390

[92] Bertran T Brachet P Vareille-Delarbre M Falenta J Dosgilbert A Vasson MP. Slight pro-inflammatory immunomodulation properties of dendritic cells by gardnerella vaginalis: the invisible man of bacterial vaginosis? J Immunol Res. (2016) 2016:9747480. 10.1155/2016/9747480 26989700 PMC4773579

[93] Moyes DL Murciano C Runglall M Islam A Thavaraj S Naglik JR. *Candida albicans* yeast and hyphae are discriminated by MAPK signaling in vaginal epithelial cells PLoS ONE. (2011) 6:e26580.e1–e9. 10.1371/journal.pone.0026580 22087232 PMC3210759

[94] Gabrielli E Sabbatini S Roselletti E Kasper L Perito S Hube B. *In vivo* induction of neutrophil chemotaxis by secretory aspartyl proteinases of *Candida albicans*. Virulence. (2016) 7:819–25. 10.1080/21505594.2016.1184385 27127904 PMC5029300

[95] Moyes DL Wilson D Richardson JP Mogavero S Tang SX Wernecke J. Candidalysin is a fungal peptide toxin critical for mucosal infection. Nature. (2016) 532:64. 10.1038/nature17625 27027296 PMC4851236

[96] Richardson JP Willems HM Moyes DL Shoaie S Barker KS Tan SL Candidalysin drives epithelial signaling, neutrophil recruitment, and immunopathology at the vaginal mucosa. Infect Immun. (2017) 2017:IAI–00645. 10.1128/IAI.00645-17 PMC5778364 29109176

[97] Moyes DL Runglall M Murciano C Shen C Nayar D Thavaraj S. A biphasic innate immune MAPK response discriminates between the yeast and hyphal forms of *Candida albicans* in epithelial cells. Cell Host Microbe. (2010) 8:225–35. 10.1016/j.chom.2010.08.002 20833374 PMC2991069

[98] Yano J Lilly E Barousse M Fidel PL. Epithelial cell-derived S100 calcium-binding proteins as key mediators in the hallmark acute neutrophil response during Candida vaginitis. Infect Immun. (2010) 78:5126–37. 10.1128/IAI.00388-10 20823201 PMC2981313

[99] Yano J Kolls JK Happel KI Wormley F Wozniak KL Fidel PL Jr. The acute neutrophil response mediated by S100 alarmins during vaginal Candida infections is independent of the Th17-pathway. PLoS ONE. (2012) 7:e46311. 10.1371/journal.pone.0046311 23050010 PMC3457984

[100] Yano J Palmer GE Eberle KE Peters BM Vogl T McKenzie AN Vaginal epithelial cell-derived S100 alarmins induced by Candida albicans via pattern recognition receptor interactions are sufficient but not necessary for the acute neutrophil response during experimental vaginal candidiasis. Infection and immunity. (2014) 82:783–92. 10.1128/IAI.00861-13 24478092 PMC3911366

[101] Weindl G Naglik JR Kaesler S Biedermann T Hube B Korting HC. Human epithelial cells establish direct antifungal defense through TLR4-mediated signaling. J Clin Invest. (2007) 117:3664–72. 10.1172/JCI28115 17992260 PMC2066194

[102] Van Asbeck EC Hoepelman AI Scharringa J Herpers BL Verhoef J. Mannose binding lectin plays a crucial role in innate immunity against yeast by enhanced complement activation and enhanced uptake of polymorphonuclear cells. BMC Microbiol. (2008) 8:229. 10.1186/1471-2180-8-229 19094203 PMC2627907

[103] Duhring S Germerodt S Skerka C Zipfel PF Dandekar T Schuster S. Host-pathogen interactions between the human innate immune system and Candida albicans—understanding and modeling defense and evasion strategies. Front Microbiol. (2015) 6:625. 10.3389/fmicb.2015.00625 26175718 PMC4485224

[104] Ip WK Lau YL. Role of mannose-binding lectin in the innate defense against *Candida albicans*: enhancement of complement activation, but lack of opsonic function, in phagocytosis by human dendritic cells. J Infect Dis. (2004) 190:632–40. 10.1086/422397 15243942

[105] Henic E Thiel S Mårdh PA. Mannan-binding lectin in women with a history of recurrent vulvovaginal candidiasis. Eur J Obstetrics Gynecol Reproduc Biol. (2010) 148:163–5. 10.1016/j.ejogrb.2009.10.008 19910100

[106] Ghazanfari M Falahati M Fattahi A Bazrafshan F Nami S Hosseinzadeh M Is MBL serum concentration a reliable predictor for recurrent vulvovaginal candidiasis? Mycoses. (2017) 2017:12723 10.1111/myc.12723 29110332

[107] Babula O Lazdane G Kroica J Ledger WJ Witkin SS. Relation between recurrent vulvovaginal candidiasis, vaginal concentrations of mannose-binding lectin, and a mannose-binding lectin gene polymorphism in Latvian women. Clin Infect Dis. (2003) 37:733–7. 10.1086/377234 12942410

[108] Liu F Liao Q Liu Z. Mannose-binding lectin and vulvovaginal candidiasis. Int J Gynecol Obstetrics. (2006) 92:43–7. 10.1016/j.ijgo.2005.08.024 16256117

[109] Urban CF Reichard U Brinkmann V Zychlinsky A. Neutrophil extracellular traps capture and kill Candida albicans yeast and hyphal forms. Cell Microbiol. (2006) 8:668–76. 10.1111/j.1462-5822.2005.00659.x 16548892

[110] Urban CF Ermert D Schmid M Abu-Abed U Goosmann C Nacken W. Neutrophil extracellular traps contain calprotectin, a cytosolic protein complex involved in host defense against *Candida albicans*. PLoS Pathog. (2009) 5:e1000639. 10.1371/journal.ppat.1000639 19876394 PMC2763347

[111] Branzk N Lubojemska A Hardison SE Wang Q Gutierrez MG Brown GD. Neutrophils sense microbe size and selectively release neutrophil extracellular traps in response to large pathogens. Nat Immunol. (2014) 15:1017. 10.1038/ni.2987 25217981 PMC4236687

[112] Kenno S Perito S Mosci P Vecchiarelli A Monari C. Autophagy and reactive oxygen species are involved in neutrophil extracellular traps release induced by *C. albicans* morphotypes. Front Microbiol. (2016) 7:879. 10.3389/fmicb.2016.00879 27375599 PMC4896927

[113] Kenny EF Herzig A Krüger R Muth A Mondal S Thompson PR. Diverse stimuli engage different neutrophil extracellular trap pathways. Elife. (2017) 6:e24437. 10.7554/eLife.24437 28574339 PMC5496738

[114] Byrd AS O'Brien XM Johnson CM Lavigne LM Reichner JS. An extracellular matrix–based mechanism of rapid neutrophil extracellular trap formation in response to *Candida albicans*. J Immunol. (2013) 190:4136–48. 10.4049/jimmunol.1202671 23509360 PMC3622194

[115] Nani S Fumagalli L Sinha U Kamen L Scapini P Berton G. Src family kinases and Syk are required for neutrophil extracellular trap formation in response to β-glucan particles. J Innate Immunity. (2015) 7:59–73. 10.1159/000365249 25277753 PMC6951106

[116] Fidel PL Barousse M Espinosa T Ficarra M Sturtevant J Martin DH. An intravaginal live Candida challenge in humans leads to new hypotheses for the immunopathogenesis of vulvovaginal candidiasis. Infect Immunity. (2004) 72:2939–46. 10.1128/IAI.72.5.2939-2946.2004 15102806 PMC387876

[117] Black CA Eyers FM Russell A Dunkley ML Clancy RL Beagley KW. Acute neutropenia decreases inflammation associated with murine vaginal candidiasis but has no effect on the course of infection. Infect Immunity. (1998) 66:1273–5. 9488427 10.1128/iai.66.3.1273-1275.1998 PMC108047

[118] Peters BM Palmer GE Nash AK Lilly EA Fidel PL Noverr MC. Fungal morphogenetic pathways are required for the hallmark inflammatory response during *Candida albicans* vaginitis. Infect Immunity. (2014) 82:532–43. 10.1128/IAI.01417-13 24478069 PMC3911367

[119] Yano J Peters BM Noverr MC Fidel PL Novel mechanism behind the immunopathogenesis of vulvovaginal candidiasis:neutrophil anergy. Infect Immunity. (2018) 86:e00684–17. 10.1128/IAI.00684-17 PMC5820946 29203543

[120] Miramon P Kasper L Hube B. Thriving within the host: *Candida* spp. interactions with phagocytic cells. Med Microbiol Immunol. (2013) 202:183–95. 10.1007/s00430-013-0288-z 23354731

[121] Cheng SC Joosten LA Kullberg BJ Netea MG. Interplay between *Candida albicans* and the mammalian innate host defense. Infect Immunity. (2012) 80:1304–13. 10.1128/IAI.06146-11 22252867 PMC3318407

[122] Wellington M Koselny K Sutterwala FS Krysan DJ. *Candida albicans* triggers NLRP3-mediated pyroptosis in macrophages. Eukaryotic Cell. (2014) 13:329–40. 10.1128/EC.00336-13 24376002 PMC3910967

[123] Uwamahoro N Verma-Gaur J Shen HH Qu Y Lewis R Lu J. The pathogen *Candida albicans* hijacks pyroptosis for escape from macrophages. MBio. (2014) 5:e00003–14. 10.1128/mBio.00003-14 24667705 PMC3977349

[124] van de Veerdonk FL Joosten LA Shaw PJ Smeekens SP Malireddi RK The inflammasome drives protective Th1 and Th17 cellular responses in disseminated candidiasis. Eur J Immunol. (2011) 41:2260–8. 10.1002/eji.201041226 21681738 PMC3939807

[125] LeibundGut-Landmann S Groß O Robinson MJ Osorio F Slack EC Tsoni SV. Syk-and CARD9-dependent coupling of innate immunity to the induction of T helper cells that produce interleukin 17. Nat Immunol. (2007) 8:630. 10.1038/ni1460 17450144

[126] Bishu S Hernández-Santos N Simpson-Abelson MR Huppler AR Conti HR Ghilardi N The adaptor CARD9 is required for adaptive but not innate immunity to oral mucosal *Candida albicans* infections. Infect Immunity. (2014) 82:1173–80. 10.1128/IAI.01335-13 24379290 PMC3958019

[127] Carvalho A Giovannini G De Luca A D'angelo C Casagrande A Iannitti RG. Dectin-1 isoforms contribute to distinct Th1/Th17 cell activation in mucosal candidiasis. Cell Mol Immunol. (2012) 9:276. 10.1038/cmi.2012.1 22543832 PMC4012853

[128] Heinsbroek SE Taylor PR Rosas M Willment JA Williams DL Gordon S. Expression of functionally different dectin-1 isoforms by murine macrophages. J Immunol. (2006) 176:5513–8. 10.4049/jimmunol.176.9.5513 16622020

[129] Ferwerda B Ferwerda G Plantinga TS Willment JA van Spriel AB Venselaar H. Human dectin-1 deficiency and mucocutaneous fungal infections. N Engl J Med. (2009) 361:1760–7. 10.1056/NEJMoa0901053 19864674 PMC2773015

[130] Li D Dong B Tong Z Wang Q Liu W Wang Y. MBL-mediated opsonophagocytosis of Candida albicans by human neutrophils is coupled with intracellular Dectin-1-triggered ROS production. PLoS ONE. (2012) 7:e50589. 10.1371/journal.pone.0050589 23239982 PMC3519760

[131] LeBlanc DM Barousse MM Fidel PL. Role for dendritic cells in immunoregulation during experimental vaginal candidiasis. Infect Immunity. (2006) 74:3213–21. 10.1128/IAI.01824-05 16714548 PMC1479243

[132] De Bernardis F Lucciarini R Boccanera M Amantini C Arancia S Morrone S. Phenotypic and functional characterization of vaginal dendritic cells in a rat model of *Candida albicans* vaginitis. Infect Immunity. (2006) 74:4282–94. 10.1128/IAI.01714-05 16790803 PMC1489681

[133] Conti HR Bruno VM Childs EE Daugherty S Hunter JP Mengesha BG. IL-17 receptor signaling in oral epithelial cells is critical for protection against oropharyngeal candidiasis. Cell Host Microbe. (2016) 20:606–17. 10.1016/j.chom.2016.10.001 27923704 PMC5147498

[134] Tomalka J Azodi E Narra HP Patel K O'Neill S Cardwell C. β-Defensin 1 plays a role in acute mucosal defense against *Candida albicans*. J Immunol. (2015) 194:1788–95. 10.4049/jimmunol.1203239 25595775 PMC4323878

[135] Tati S Davidow P McCall A Hwang-Wong E Rojas IG Cormack B. Candida glabrata binding to *Candida albicans* hyphae enables its development in oropharyngeal candidiasis. PLoS Pathog. (2016) 12:e1005522. 10.1371/journal.ppat.1005522 27029023 PMC4814137

[136] Nawrot U Grzybek-Hryncewicz K Zielska U Czarny A Podwinska J. The study of cell-mediated immune response in recurrent vulvovaginal candidiasis. FEMS Immunol Med Microbiol. (2000) 29:89–94. 10.1111/j.1574-695X.2000.tb01509.x 11024346

[137] Pietrella D Rachini A Pines M Pandey N Mosci P Bistoni F. Th17 cells and IL-17 in protective immunity to vaginal candidiasis. PLoS ONE. (2011) 6:e22770. 10.1371/journal.pone.0022770 21818387 PMC3144947

[138] Talaei Z Sheikhbahaei S Ostadi V Hakemi MG Meidani M Naghshineh E. Recurrent vulvovaginal Candidiasis: could it be related to cell-mediated immunity defect in response to Candida antigen? Int J Fertility Sterility. (2017) 11:134. 10.22074/ijfs.2017.4883 28868834 PMC5582140

[139] Fidel PL Jr Lynch ME Lopez VR Sobel JD Robinson R. Systemic cell-mediated immune reactivity in women with recurrent vulvovaginal candidiasis. J Infect Dis. (1993) 168:1458–65. 10.1093/infdis/168.6.1458 8245529

[140] Mendling W Koldovsky U. Investigations by cell-mediated immunologic tests and therapeutic trials with thymopentin in vaginal mycoses. Infect Dis Obstetr Gynecol. (1996) 4:225–31. 18476097 10.1155/S1064744996000439 PMC2364501

[141] Cassone A Boccanera M Adriani DANIELA Santoni G De Bernardis FLAVIA. Rats clearing a vaginal infection by *Candida albicans* acquire specific, antibody-mediated resistance to vaginal reinfection. Infect Immunity. (1995) 63:2619–24. 7790077 10.1128/iai.63.7.2619-2624.1995 PMC173351

[142] De Bernardis FLAVIA Boccanera M Adriani D Spreghini E Santoni G Cassone A. Protective role of antimannan and anti-aspartyl proteinase antibodies in an experimental model of *Candida albicans* vaginitis in rats. Infect Immunity. (1997) 65:3399–405. 9234804 10.1128/iai.65.8.3399-3405.1997 PMC175481

[143] De Bernardis F Santoni G Boccanera M Spreghini E Adriani D Morelli L. Local anticandidal immune responses in a rat model of vaginal infection by and protection against *Candida albicans*. Infect Immunity. (2000) 68:3297–304. 10.1128/IAI.68.6.3297-3304.2000 10816477 PMC97585

[144] Spits H Artis D Colonna M Diefenbach A Di Santo JP Eberl G. Innate lymphoid cells—a proposal for uniform nomenclature. Nat Rev Immunol. (2013) 13:145. 10.1038/nri3365 23348417

[145] Conti HR Gaffen SL. IL-17–Mediated immunity to the opportunistic fungal pathogen *Candida albicans*. J Immunol. (2015) 195:780–8. 10.4049/jimmunol.1500909 26188072 PMC4507294

[146] Isailovic N Daigo K Mantovani A Selmi C. Interleukin-17 and innate immunity in infections and chronic inflammation. J Autoimmunity. (2015) 60:1–11. 10.1016/j.jaut.2015.04.006 25998834

[147] Ferretti S Bonneau O Dubois GR Jones CE Trifilieff A. IL-17, produced by lymphocytes and neutrophils, is necessary for lipopolysaccharide-induced airway neutrophilia: IL-15 as a possible trigger. J Immunol. (2003) 170:2106–12. 10.4049/jimmunol.170.4.2106 12574382

[148] Huppler AR Verma AH Conti HR Gaffen SL Neutrophils do not express IL-17A in the context of acute oropharyngeal candidiasis. Pathogens. (2015) 4:559–72. 10.3390/pathogens4030559 26213975 PMC4584272

[149] Verma AH Richardson JP Zhou C Coleman BM Moyes DL Ho J. Oral epithelial cells orchestrate innate type 17 responses to *Candida albicans* through the virulence factor candidalysin. Sci Immunol. (2017) 2:eaam8834. 10.1126/sciimmunol.aam8834 29101209 PMC5881387

[150] Gillin FD Sher ALAN. Activation of the alternative complement pathway by *Trichomonas vaginalis*. Infect Immunity. (1981) 34:268–73. 6795124 10.1128/iai.34.1.268-273.1981 PMC350852

[151] Shaio MF Chang FY Hou SC Lee CS Lin PR. The role of immunoglobulin and complement in enhancing the respiratory burst of neutrophils against *Trichomonas vaginalis*. Parasite Immunol. (1991) 13:241–50. 10.1111/j.1365-3024.1991.tb00279.x 1852474

[152] Malla N Goyal K Dhanda RS Yadav M. Immunity in urogenital protozoa. Parasite Immunol. (2014) 36:400–8. 10.1111/pim.12114 25201404

[153] Chatterjee A Ratner DM Ryan CM Johnson PJ O'Keefe BR Secor WE. Anti-retroviral lectins have modest effects on adherence of trichomonas vaginalis to epithelial cells *in vitro* and on recovery of Tritrichomonas foetus in a mouse vaginal model. PLoS ONE. (2015) 10:e0135340. 10.1371/journal.pone.0135340 26252012 PMC4529277

[154] Menezes CB Tasca T. Trichomoniasis immunity and the involvement of the purinergic signaling. Biomed J. (2016) 39:234–43. 10.1016/j.bj.2016.06.007 27793265 PMC6138788

[155] Zariffard MR Harwani S Novak RM Graham PJ Ji X Spear GT. Trichomonas vaginalis infection activates cells through toll-like receptor 4. Clin Immunol. (2004) 111:103–7. 10.1016/j.clim.2003.12.008 15093558

[156] Chang JH Park JY Kim SK. Dependence on p38 MAPK signalling in the up-regulation of TLR2, TLR4 and TLR9 gene expression in Trichomonas vaginalis-treated HeLa cells. Immunology. (2006) 118:164–70. 10.1111/j.1365-2567.2006.02347.x 16771851 PMC1782292

[157] Fichorova RN Trifonova RT Gilbert RO Costello CE Hayes GR Lucas JJ. Trichomonas vaginalis lipophosphoglycan triggers a selective upregulation of cytokines by human female reproductive tract epithelial cells. Infect Immunity. (2006) 74:5773–9. 10.1128/IAI.00631-06 16988255 PMC1594934

[158] Okumura CY Baum LG Johnson PJ. Galectin-1 on cervical epithelial cells is a receptor for the sexually transmitted human parasite Trichomonas vaginalis. Cell Microbiol. (2008) 10:2078–90. 10.1111/j.1462-5822.2008.01190.x 18637021 PMC4437540

[159] Fichorova RN Yamamoto HS Fashemi T Foley E Ryan S Beatty N. *Trichomonas vaginalis* lipophosphoglycan exploits binding to galectin-1 and-3 to modulate epithelial immunity. J Biol Chem. (2016) 291:998–1013. 10.1074/jbc.M115.651497 26589797 PMC4705417

[160] Nam YH Min D Kim HP Song KJ Kim KA Lee YA. Leukotriene B4 receptor BLT-mediated phosphorylation of NF-κB and CREB is involved in IL-8 production in human mast cells induced by Trichomonas vaginalis-derived secretory products. Microbes Infect. (2011) 13:1211–20. 10.1016/j.micinf.2011.07.006 21824526

[161] Nam YH Min A Kim SH Lee YA Kim KA Song KJ Leukotriene B4 receptors BLT1 and BLT2 are involved in interleukin-8 production in human neutrophils induced by *Trichomonas vaginalis*-derived secretory products. Inflamm Res. (2012) 61:97–102. 10.1007/s00011-011-0425-3 22215047

[162] Le Bel M Brunet A Gosselin J. Leukotriene B4, an endogenous stimulator of the innate immune response against pathogens. J Innate Immunity. (2014) 6:159–68. 10.1159/000353694 23988515 PMC6741447

[163] Song HO Shin MH Ahn MH Min DY Kim YS Ryu JS. Trichomonas vaginalis: reactive oxygen species mediates caspase-3 dependent apoptosis of human neutrophils. Exp Parasitol. (2008) 118:59–65. 10.1016/j.exppara.2007.06.010 17709105

[164] Ryu JS Kang JH Jung SY Shin MH Kim JM Park H. Production of interleukin-8 by human neutrophils stimulated with *Trichomonas vaginalis*. Infect Immunity. (2004) 72:1326–32. 10.1128/IAI.72.3.1326-1332.2004 14977935 PMC355987

[165] Han IH Goo SY Park SJ Hwang SJ Kim YS Yang MS. Proinflammatory cytokine and nitric oxide production by human macrophages stimulated with *Trichomonas vaginalis*. Kor J Parasitol. (2009) 47:205. 10.3347/kjp.2009.47.3.205 19724692 PMC2735684

[166] Mercer F Ng SH Brown TM Boatman G Johnson PJ. Neutrophils kill the parasite *Trichomonas vaginalis* using trogocytosis. PLoS Biol. (2018) 16:e2003885. 10.1371/journal.pbio.2003885 29408891 PMC5815619

[167] Huppert JS Huang B Chen C Dawood HY Fichorova RN. Clinical evidence for the role of *Trichomonas vaginalis* in regulation of secretory leukocyte protease inhibitor in the female genital tract. J Infect Dis. (2013) 207:1462–70. 10.1093/infdis/jit039 23355743 PMC3610423

[168] Smith JD Garber GE. *Trichomonas vaginalis* infection induces vaginal CD4+ T-Cell infiltration in a mouse model: a vaccine strategy to reduce vaginal infection and HIV transmission. J Infect Dis. (2015) 212:285–93. 10.1093/infdis/jiv036 25616405

[169] Malla N Yadav M Gupta I. Kinetics of serum and local cytokine profile in experimental intravaginal trichomoniasis induced with *Trichomonas vaginalis* isolates from symptomatic and asymptomatic women. Parasite Immunol. (2007) 29:101–5. 10.1111/j.1365-3024.2006.00914.x 17241398

[170] Makinde HM Zariffard R Mirmonsef P Novak RM Jarrett O Landay AL. IL-22 levels are associated with *Trichomonas vaginalis* infection in the lower genital tract. Am J Reproduc Immunol. (2013) 70:38–44. 10.1111/aji.12100 23445169 PMC3675182

[171] Dempsey LA. Antimicrobial IL-22. Nat Immunol. (2017) 18:373–373. 10.1038/ni.3722 28323264

[172] Eyerich K Dimartino V Cavani A. IL-17 and IL-22 in immunity: driving protection and pathology. Eur J Immunol. (2017) 47:607–14. 10.1002/eji.201646723 28295238

[173] Zhang Y Zhang Y Gu W Sun B TH1/TH2 cell differentiation and molecular signals. In: Sun B, editor. T Helper Cell Differentiation and Their Function. Dordrecht: Springer (2014). p. 15–44. 10.1007/978-94-017-9487-9_2

[174] Yadav M Gupta I Malla N. Kinetics of immunoglobulin G, M, A and IgG subclass responses in experimental intravaginal trichomoniasis: prominence of IgG1 response. Parasite Immunol. (2005) 27:461–7. 10.1111/j.1365-3024.2005.00800.x 16255745

[175] Kaur S Khurana S Bagga R Wanchu A Malla N Trichomoniasis among women in North India: A hospital based study. Indian J Sexually Trans Dis AIDS. (2008) 29:76 10.4103/0253-7184.48729

[176] Bastida-Corcuera FD Singh BN Gray GC Stamper PD Davuluri M Schlangen K Antibodies to *Trichomonas vaginalis* surface glycolipid. Sex Transm Infect. (2013) 2013:sextrans–2012. 10.1136/sextrans-2012-051013 PMC4685936 23785040

[177] Nu PAT Nguyen VQH Cao NT Dessì D Rappelli P Fiori PL Prevalence of *Trichomonas vaginalis* infection in symptomatic and asymptomatic women in Central Vietnam. J Infect Dev Countries. (2015) 9:655–60. 10.3855/jidc.7190 26142677

[178] Provenzano D Alderete JF. Analysis of human immunoglobulin-degrading cysteine proteinases of *Trichomonas vaginalis*. Infect Immunity. (1995) 63:3388–95. 7642267 10.1128/iai.63.9.3388-3395.1995 PMC173466

[179] Yadav M Dubey ML Gupta I Malla N. Cysteine proteinase 30 (CP30) and antibody response to CP30 in serum and vaginal washes of symptomatic and asymptomatic *Trichomonas vaginalis*-infected women. Parasite Immunol. (2007) 29:359–65. 10.1111/j.1365-3024.2007.00952.x 17576365

[180] Birben E Sahiner UM Sackesen C Erzurum S Kalayci O. Oxidative stress and antioxidant defense. World Allergy Organ J. (2012) 5:9. 10.1097/WOX.0b013e3182439613 23268465 PMC3488923

[181] Lugrin J Rosenblatt-Velin N Parapanov R Liaudet L. The role of oxidative stress during inflammatory processes. Biol Chem. (2014) 395:203–30. 10.1515/hsz-2013-0241 24127541

[182] Pacher P Beckman JS Liaudet L. Nitric oxide and peroxynitrite in health and disease. Physiol Rev. (2007) 87:315–424. 10.1152/physrev.00029.2006 17237348 PMC2248324

[183] Jomova K Vondrakova D Lawson M Valko M. Metals, oxidative stress and neurodegenerative disorders. Mol Cell Biochem. (2010) 345:91–104. 10.1007/s11010-010-0563-x 20730621

[184] Forstermann U Sessa WC. Nitric oxide synthases: regulation and function. Eur Heart J. (2011) 33:829–37. 10.1093/eurheartj/ehr304 21890489 PMC3345541

[185] Tsung A Klune JR Zhang X Jeyabalan G Cao Z Peng X. HMGB1 release induced by liver ischemia involves Toll-like receptor 4–dependent reactive oxygen species production and calcium-mediated signaling. J Exp Med. (2007) 204:2913–23. 10.1084/jem.20070247 17984303 PMC2118528

[186] Tang D Kang R Livesey KM Zeh III HJ Lotze MT. High mobility group box 1 (HMGB1) activates an autophagic response to oxidative stress. Antioxid Redox Signal. (2011) 15:2185–95. 10.1089/ars.2010.3666 21395369 PMC3166205

[187] Loukili N Rosenblatt-Velin N Li J Clerc S Pacher P Feihl F. Peroxynitrite induces HMGB1 release by cardiac cells *in vitro* and HMGB1 upregulation in the infarcted myocardium *in vivo*. Cardiovasc Res. (2010) 89:586–94. 10.1093/cvr/cvq373 21113057 PMC3028979

[188] Fan J Li Y Levy RM Fan JJ Hackam DJ Vodovotz Y. Hemorrhagic shock induces NAD (P) H oxidase activation in neutrophils: role of HMGB1-TLR4 signaling. J Immunol. (2007) 178:6573–80. 10.4049/jimmunol.178.10.6573 17475888

[189] Janko C Filipović M Munoz LE Schorn C Schett G Ivanović-Burmazović I. Redox modulation of HMGB1-related signaling. Antioxid Redox Signal. (2014) 20:1075–85. 10.1089/ars.2013.5179 23373897 PMC3928832

[190] Powers KA Szászi K Khadaroo RG Tawadros PS Marshall JC Kapus A. Oxidative stress generated by hemorrhagic shock recruits Toll-like receptor 4 to the plasma membrane in macrophages. J Exp Med. (2006) 203:1951–61. 10.1084/jem.20060943 16847070 PMC2118368

[191] Nakahira K Kim HP Geng XH Nakao A Wang X Murase N. Carbon monoxide differentially inhibits TLR signaling pathways by regulating ROS-induced trafficking of TLRs to lipid rafts. J Exp Med. (2006) 203:2377–89. 10.1084/jem.20060845 17000866 PMC2118097

[192] Gill R Tsung A Billiar T. Linking oxidative stress to inflammation: toll-like receptors. Free Radical Biol Med. (2010) 48:1121–32. 10.1016/j.freeradbiomed.2010.01.006 20083193 PMC3423196

[193] Lucas K Maes M. Role of the Toll Like receptor (TLR) radical cycle in chronic inflammation: possible treatments targeting the TLR4 pathway. Mol Neurobiol. (2013) 48:190–204. 10.1007/s12035-013-8425-7 23436141 PMC7091222

[194] Gloire G Legrand-Poels S Piette J. NF-κB activation by reactive oxygen species: fifteen years later. Biochem Pharmacol. (2006) 72:1493–505. 10.1016/j.bcp.2006.04.011 16723122

[195] Tschopp J Schroder K. NLRP3 inflammasome activation: the convergence of multiple signalling pathways on ROS production? Nat Rev Immunol. (2010) 10:210. 10.1038/nri2725 20168318

[196] Shimada K Crother TR Karlin J Dagvadorj J Chiba N Chen S. Oxidized mitochondrial DNA activates the NLRP3 inflammasome during apoptosis. Immunity. (2012) 36:401–14. 10.1016/j.immuni.2012.01.009 22342844 PMC3312986

[197] Li H Horke S Förstermann U. Oxidative stress in vascular disease and its pharmacological prevention. Trends Pharmacol Sci. (2013) 34:313–9. 10.1016/j.tips.2013.03.007 23608227

[198] Simhan HN Anderson BL Krohn MA Heine RP de Tejada BM Landers DV. Host immune consequences of asymptomatic *Trichomonas vaginalis* infection in pregnancy. Am J Obstetr Gynecol. (2007) 196:59–e1. 10.1016/j.ajog.2006.08.035 17240235

[199] Escario A Barrio AG Diez BS Escario JA. Immunohistochemical study of the vaginal inflammatory response in experimental trichomoniasis. Acta Tropica. (2010) 114:22–30. 10.1016/j.actatropica.2009.12.002 20025844

[200] Frasson AP De Carli GA Bonan CD Tasca T. Involvement of purinergic signaling on nitric oxide production by neutrophils stimulated with *Trichomonas vaginalis*. Puriner Signal. (2012) 8:1–9. 10.1007/s11302-011-9254-7 21833696 PMC3286535

[201] Svobodová E Staib P Losse J Hennicke F Barz D Józsi M. Differential interaction of the two related fungal species *Candida albicans* and *Candida dubliniensis* with human neutrophils. J Immunol. (2012) 189:2502–11. 10.4049/jimmunol.1200185 22851712

[202] Johnson CJ Kernien JF Hoyer AR Nett JE. Mechanisms involved in the triggering of neutrophil extracellular traps (NETs) by *Candida glabrata* during planktonic and biofilm growth. Sci Rep. (2017) 7:13065. 10.1038/s41598-017-13588-6 29026191 PMC5638821

[203] Chen Z Zhang Z Zhang H Xie B. Analysis of the oxidative stress status in nonspecific vaginitis and its role in vaginal epithelial cells apoptosis. BioMed Res Int. (2015) 2015:795656. 10.1155/2015/795656 26558281 PMC4628999

[204] de Souza Bonfim-Mendonça P Ratti BA Negri M Lima NC Fiorini A Hatanaka E β-Glucan induces reactive oxygen species production in human neutrophils to improve the killing of *Candida albicans* and *Candida glabrata* isolates from vulvovaginal candidiasis. PLoS ONE. (2014) 9:e107805 10.1371/journal.pone.0107805 25229476 PMC4168232

[205] Babula O Lazdāne G Kroica J Linhares IM Ledger WJ Witkin SS. Frequency of interleukin-4 (IL-4)-589 gene polymorphism and vaginal concentrations of IL-4, nitric oxide, and mannose-binding lectin in women with recurrent vulvovaginal candidiasis. Clin Infect Dis. (2005) 40:1258–62. 10.1086/429246 15825027

[206] Kalia N Kaur M Sharma S Singh J Impaired PRR expression modulates inflammation-triggered oxidative stress and pathogenesis of recurrent vulvovaginal infections. Bull Natl Res Centre. (2019) 43:109 10.1186/s42269-019-0147-1

[207] Gun-Chas PARK RYE JS Duh-Young MIN The role of nitric oxide as an effector of macrophage—mediated cytotoxicity against ‘I ‘richomonas vaginalis. Kor J Parasitol. (1997) 35:189–95. 10.3347/kjp.1997.35.3.189 9335184

[208] Malla N Valadkhani Z Harjai K Sharma S Gupta I. Reactive nitrogen intermediates in experimental trichomoniasis induced with isolates from symptomatic and asymptomatic women. Parasitol Res. (2004) 94:101–5. 10.1007/s00436-004-1155-z 15309620

[209] Yadav M Dubey ML Gupta I Malla N. Nitric oxide radicals in leucocytes and vaginal washes of *Trichomonas vaginalis*-infected symptomatic and asymptomatic women. Parasitology. (2006) 132:339–43. 10.1017/S0031182005009340 16529664

[210] Okin D Medzhitov R. Evolution of inflammatory diseases. Curr Biol. (2012) 22:R733–40. 10.1016/j.cub.2012.07.029 22975004 PMC3601794

[211] Aldunate M Srbinovski D Hearps AC Latham CF Ramsland PA Gugasyan R. Antimicrobial and immune modulatory effects of lactic acid and short chain fatty acids produced by vaginal microbiota associated with eubiosis and bacterial vaginosis. Front Physiol. (2015) 6:164. 10.3389/fphys.2015.00164 26082720 PMC4451362

[212] Harvey HA Swords WE Apicella MA. The mimicry of human glycolipids and glycosphingolipids by the lipooligosaccharides of pathogenic neisseria and haemophilus. J Autoimmunity. (2001) 16:257–62. 10.1006/jaut.2000.0477 11334490

[213] Severi E Hood DW Thomas GH. Sialic acid utilization by bacterial pathogens. Microbiology. (2007) 153:2817–22. 10.1099/mic.0.2007/009480-0 17768226

[214] Varki A Gagneux P. Multifarious roles of sialic acids in immunity. Ann N Y Acad Sci. (2012) 1253:16–36. 10.1111/j.1749-6632.2012.06517.x 22524423 PMC3357316

[215] Gantner BN Simmons RM Underhill DM Dectin-1 mediates macrophage recognition of *Candida albicans* yeast but not filaments. EMBO J. (2005) 24:1277–86. 10.1038/sj.emboj.7600594 15729357 PMC556398

[216] Gropp K Schild L Schindler S Hube B Zipfel PF Skerka C. The yeast *Candida albicans* evades human complement attack by secretion of aspartic proteases. Mol Immunol. (2009) 47:465–75. 10.1016/j.molimm.2009.08.019 19880183

[217] Meri T Hartmann A Lenk D Eck R Würzner R Hellwage J. The yeast *Candida albicans* binds complement regulators factor H and FHL-1. Infect Immun. (2002) 70:5185–92. 10.1128/IAI.70.9.5185-5192.2002 12183569 PMC128257

[218] Meri T Blom AM Hartmann A Lenk D Meri S Zipfel PF. The hyphal and yeast forms of Candida albicans bind the complement regulator C4b-binding protein. Infect Immun. (2004) 72:6633–41. 10.1128/IAI.72.11.6633-6641.2004 15501796 PMC523010

[219] Poltermann S Kunert A von der Heide M Eck R Hartmann A Zipfel PF. Gpm1p is a factor H-, FHL-1-, and plasminogen-binding surface protein of *Candida albicans*. J Biol Chem. (2007) 282:37537–44. 10.1074/jbc.M707280200 17959597

[220] Luo S Poltermann S Kunert A Rupp S Zipfel PF. Immune evasion of the human pathogenic yeast *Candida albicans*: Pra1 is a Factor H, FHL-1 and plasminogen binding surface protein. Mol Immunol. (2009) 47:541–50. 10.1016/j.molimm.2009.07.017 19850343

[221] Luo S Hartmann A Dahse HM Skerka C Zipfel PF. Secreted pH-regulated antigen 1 of *Candida albicans* blocks activation and conversion of complement C3. J Immunol. (2010) 185:2164–73. 10.4049/jimmunol.1001011 20644161

[222] Luo S Blom AM Rupp S Hipler UC Hube B Skerka C. The pH-regulated antigen 1 of *Candida albicans* binds the human complement inhibitor C4b-binding protein and mediates fungal complement evasion. J Biol Chem. (2011) 286:8021–9. 10.1074/jbc.M110.130138 21212281 PMC3048689

[223] Fernández-Arenas E Bleck CK Nombela C Gil C Griffiths G Diez-Orejas R. *Candida albicans* actively modulates intracellular membrane trafficking in mouse macrophage phagosomes. Cell Microbiol. (2009) 11:560–89. 10.1111/j.1462-5822.2008.01274.x 19134116

[224] McKenzie CGJ Koser U Lewis LE Bain JM Mora-Montes HM Barker RN. Contribution of *Candida albicans* cell wall components to recognition by and escape from murine macrophages. Infect Immunity. (2010) 78:1650–8. 10.1128/IAI.00001-10 20123707 PMC2849426

[225] O'Meara TR Veri AO Ketela T Jiang B Roemer T Cowen LE. Global analysis of fungal morphology exposes mechanisms of host cell escape. Nat Commun. (2015) 6:6741. 10.1038/ncomms7741 25824284 PMC4382923

[226] Frohner IE Bourgeois C Yatsyk K Majer O Kuchler K. *Candida albicans* cell surface superoxide dismutases degrade host-derived reactive oxygen species to escape innate immune surveillance. Mol Microbiol. (2009) 71:240–52. 10.1111/j.1365-2958.2008.06528.x 19019164 PMC2713856

[227] Wellington M Dolan K Krysan DJ. Live *Candida albicans* suppresses production of reactive oxygen species in phagocytes. Infect Immunity. (2009) 77:405–13. 10.1128/IAI.00860-08 18981256 PMC2612242

[228] Nakagawa T Kawasaki N Ma Y Uemura K Kawasaki T. Antitumor activity of mannan-binding protein. Methods Enzymol. (2003) 363:26–33. 10.1016/S0076-6879(03)01041-3 14579565

[229] Palmer GE Kelly MN Sturtevant JE. The *Candida albicans* vacuole is required for differentiation and efficient macrophage killing. Eukaryotic Cell. (2005) 4:1677–86. 10.1128/EC.4.10.1677-1686.2005 16215175 PMC1265890

[230] Palmer GE. Vacuolar trafficking and *Candida albicans* pathogenesis. Commun Integr Biol. (2011) 4:240–2. 10.4161/cib.4.2.14717 21655451 PMC3104590

[231] Xiong J Kang K Liu L Yoshida Y Cooper KD Ghannoum MA. *Candida albicans* and *Candida krusei* differentially induce human blood mononuclear cell interleukin-12 and gamma interferon production. Infect Immunity. (2000) 68:2464–9. 10.1128/IAI.68.5.2464-2469.2000 10768932 PMC97447

[232] Tang N Liu L Kang K Mukherjee PK Takahara M Chen G. Inhibition of monocytic interleukin-12 production by *Candida albicans* via selective activation of ERK mitogen-activated protein kinase. Infect Immunity. (2004) 72:2513–20. 10.1128/IAI.72.5.2513-2520.2004 15102758 PMC387890

[233] Wang M Mukherjee PK Chandra J Lattif AA McCormick TS Ghannoum MA. Characterization and partial purification of *Candida albicans* secretory IL-12 inhibitory factor. BMC Microbiol. (2008) 8:31. 10.1186/1471-2180-8-31 18282300 PMC2289826

[234] Cheng SC van de Veerdonk F Smeekens S Joosten LA van der Meer JW Kullberg BJ. *Candida albicans* dampens host defense by downregulating IL-17 production. J Immunol. (2010) 185:2450–7. 10.4049/jimmunol.1000756 20624941

[235] Chang JH Ryang YS Morio T Lee SK Chang EJ. *Trichomonas vaginalis* inhibits proinflammatory cytokine production in macrophages by suppressing NF-κB activation. Mol Cells. (2004) 18:177–85. 15528993

[236] Song MJ Lee JJ Nam YH Kim TG Chung YW Kim M. Modulation of dendritic cell function by Trichomonas vaginalis-derived secretory products. BMB Rep. (2015) 48:103. 10.5483/BMBRep.2015.48.2.116 24965578 PMC4352611

[237] Alderete JF Provenzano D Lehker MW. Iron mediates *Trichomonas vaginalis* resistance to complement lysis. Microb Pathogen. (1995) 19:93–103. 10.1006/mpat.1995.0049 8577239

[238] Draper D Donohoe W Mortimer L Heine RP. Cysteine proteases of Trichomonas vaginalis degrade secretory leukocyte protease inhibitor. J Infect Dis. (1998) 178:815–9. 10.1086/515366 9728551

[239] Min DY Hyun KH Ryu JS Ahn MH Cho MH. Degradations of human immunoglobulins and hemoglobin by a 60 kDa cysteine proteinase of *Trichomonas vaginalis*. Kor J Parasitol. (1998) 36:261–68. 10.3347/kjp.1998.36.4.261 9868892 PMC2732966

[240] Alvarez-Sánchez ME Solano-González E Yañez-Gómez C Arroyo R. Negative iron regulation of the CP65 cysteine proteinase cytotoxicity in *Trichomonas vaginalis*. Microbes Infect. (2007) 9:1597–605. 10.1016/j.micinf.2007.09.011 18023389

[241] Malla N Kaul P Sehgal R Gupta I. *In vitro* haemolytic and cytotoxic activity of soluble extract antigen of *T vaginalis* isolates from symptomatic and asymptomatic women. Parasitol Res. (2008) 102:1375–78. 10.1007/s00436-008-0947-y 18369662

[242] Pereira-Neves A Benchimol M. Phagocytosis by *Trichomonas vaginalis*: new insights. Biol Cell. (2007) 99:87–101. 10.1042/BC20060084 17029588

[243] Mercer F Diala FGI Chen YP Molgora BM Ng SH Johnson PJ. Leukocyte lysis and cytokine induction by the human sexually transmitted parasite *Trichomonas vaginalis*. PLoS Negl Tropic Dis. (2016) 10:e0004913. 10.1371/journal.pntd.0004913 27529696 PMC4986988

[244] Alderete JF Garza GE. Identification and properties of *Trichomonas vaginalis* proteins involved in cytadherence. Infect Immunity. (1988) 56:28–33. 3257206 10.1128/iai.56.1.28-33.1988 PMC259228

[245] Lehker MW Alderete JF. Biology of trichomonosis. Curr Opin Infect Dis. (2000) 13:37–45. 10.1097/00001432-200002000-00007 11964771

[246] Nemati M Malla N Yadav M Khorramdelazad H Jafarzadeh A. Humoral and T cell-mediated immune response against trichomoniasis. Parasite Immunol. (2017) 40:e12510.e1–e17. 10.1111/pim.12510 29266263

[247] Peterson KM Alderete JF. Host plasma proteins on the surface of pathogenic *Trichomonas vaginalis*. Infect Immunity. (1982) 37:755–62. 6288571 10.1128/iai.37.2.755-762.1982 PMC347594

[248] Ibanez-Escribano A Nogal-Ruiz JJ Pérez-Serrano J Gómez-Barrio A Escario JA Alderete JF. Sequestration of host-CD59 as potential immune evasion strategy of *Trichomonas vaginalis*. Acta Tropic. (2015) 149:1–7. 10.1016/j.actatropica.2015.05.003 25976413

[249] Almkvist J Dahlgren C Leffler H Karlsson A. Activation of the neutrophil nicotinamide adenine dinucleotide phosphate oxidase by galectin-1. J Immunol. (2002) 168:4034–41. 10.4049/jimmunol.168.8.4034 11937561

[250] Li Y Komai-Koma M Gilchrist DS Hsu DK Liu FT Springall T. Galectin-3 is a negative regulator of lipopolysaccharide-mediated inflammation. J Immunol. (2008) 181:2781–9. 10.4049/jimmunol.181.4.2781 18684969

[251] Vasta GR Feng C González-Montalbán N Mancini J Yang L Abernathy K. Functions of galectins as ‘self/non-self'-recognition and effector factors. Pathogens Dis. (2017) 75:46. 10.1093/femspd/ftx046 28449072 PMC5808656

[252] Dam TK Brewer FC. Maintenance of cell surface glycan density by lectin–glycan interactions: a homeostatic and innate immune regulatory mechanism. Glycobiology. (2010) 20:1061–64. 10.1093/glycob/cwq084 20548106

[253] Chang JH Kim SK Choi IH Lee SK Morio T Chang EJ. Apoptosis of macrophages induced by Trichomonas vaginalis through the phosphorylation of p38 mitogen-activated protein kinase that locates at downstream of mitochondria-dependent caspase activation. Int J Biochem Cell Biol. (2006) 38:638–47. 10.1016/j.biocel.2005.11.005 16360334

[254] Kang JH Song HO Ryu JS Shin MH Kim JM Cho YS. *Trichomonas vaginalis* promotes apoptosis of human neutrophils by activating caspase-3 and reducing Mcl-1 expression. Parasite Immunol. (2006) 28:439–46. 10.1111/j.1365-3024.2006.00884.x 16916367 PMC2562650

[255] Olmos-Ortiz LM Barajas-Mendiola MA Barrios-Rodiles M Castellano LE Arias-Negrete S Avila EE. *Trichomonas vaginalis* exosome-like vesicles modify the cytokine profile and reduce inflammation in parasite-infected mice. Parasite Immunol. (2017) 39:e12426. 10.1111/pim.12426 28345149

[256] Alderete JF Demas P Gombosova A Valent MICHAL Yanoska A Fabusova H. Phenotypes and protein-epitope phenotypic variation among fresh isolates of *Trichomonas vaginalis*. Infect Immunity. (1987) 55:1037–41. 2437029 10.1128/iai.55.5.1037-1041.1987 PMC260464

[257] Alderete JF. Iron modulates phenotypic variation and phosphorylation of P270 in double-stranded RNA virus-infected Trichomonas vaginalis. Infect Immunity. (1999) 67:4298–302. 10417210 10.1128/iai.67.8.4298-4302.1999 PMC96743

[258] Genc MR Onderdonk AB Vardhana S Delaney ML Norwitz ER Tuomala RE. Polymorphism in intron 2 of the interleukin-1 receptor antagonist gene, local midtrimester cytokine response to vaginal flora, and subsequent preterm birth. Am J Obstetr Gynecol. (2004) 191:1324–30. 10.1016/j.ajog.2004.05.074 15507961

[259] Cauci S Di Santolo M Casabellata G Ryckman K Williams SM Guaschino S. Association of interleukin-1β and interleukin-1 receptor antagonist polymorphisms with bacterial vaginosis in non-pregnant Italian women. MHR. (2007) 13:243–50. 10.1093/molehr/gam002 17314118

[260] Goepfert AR Varner M Ward K Macpherson C Klebanoff M Goldenberg RL. Differences in inflammatory cytokine and Toll-like receptor genes and bacterial vaginosis in pregnancy. Am J Obstetr Gynecol. (2005) 193:1478–85. 10.1016/j.ajog.2005.03.053 16202743

[261] Gómez LM Sammel MD Appleby DH Elovitz MA Baldwin DA Jeffcoat MK. Evidence of a gene-environment interaction that predisposes to spontaneous preterm birth: a role for asymptomatic bacterial vaginosis and DNA variants in genes that control the inflammatory response. Am J Obstetr Gynecol. (2010) 202:386–e1. 10.1016/j.ajog.2010.01.042 20350647

[262] Macones GA Parry S Elkousy M Clothier B Ural SH Strauss JF. A polymorphism in the promoter region of TNF and bacterial vaginosis: preliminary evidence of gene-environment interaction in the etiology of spontaneous preterm birth. Am J Obstetr Gynecol. (2004) 190:1504–8. 10.1016/j.ajog.2004.01.001 15284722

[263] Genç MR Vardhana S Delaney ML Witkin SS Onderdonk AB. TNFA-308G> A polymorphism influences the TNF-α response to altered vaginal flora. Eur J Obstetr Gynecol Reproduc Biol. (2007) 134:188–91. 10.1016/j.ejogrb.2006.10.018 17123692

[264] Ryckman KK Simhan HN Krohn MA Williams SM. Predicting risk of bacterial vaginosis: the role of race, smoking and corticotropin-releasing hormone-related genes. Mol Hum Reproduc. (2009) 15:131–7. 10.1093/molehr/gan081 19131402 PMC2734163

[265] Fang X Li H Diao Y Shan R Dong J Li H. Polymorphisms in the MTHRF, VDR, MMP-9 and IL-β genes and the risk of premature rupture of membranes. Gynecol Obstetr Invest. (2010) 70:206–14. 10.1159/000318867 20639647

[266] Taylor BD Darville T Ferrell RE Ness RB Kelsey SF Haggerty CL Cross-sectional analysis of Toll-like receptor variants and bacterial vaginosis in African–American women with pelvic inflammatory disease. Sex Transm Infect. (2014) 2014:51524 10.1136/sextrans-2014-051524 24848367

[267] Mackelprang RD Scoville CW Cohen CR Ondondo RO Bigham AW Celum C. Toll-like receptor gene variants and bacterial vaginosis among HIV-1 infected and uninfected African women. Genes Immunity. (2015) 16:362. 10.1038/gene.2015.13 25928881 PMC4523061

[268] Royse KE Kempf MC McGwin G Jr Wilson CM Tang J Shrestha S. Toll-like receptor gene variants associated with bacterial vaginosis among HIV-1 infected adolescents. J Reproduc Immunol. (2012) 96:84–9. 10.1016/j.jri.2012.08.002 23021866 PMC3518650

[269] Rosentul DC Delsing CE Jaeger M Plantinga TS Oosting M Costantini I. Gene polymorphisms in pattern recognition receptors and susceptibility to idiopathic recurrent vulvovaginal candidiasis. Front Microbiol. (2014) 5:483. 10.3389/fmicb.2014.00483 25295030 PMC4172055

[270] Lev-Sagie A Prus D Linhares IM Lavy Y Ledger WJ Witkin SS. Polymorphism in a gene coding for the inflammasome component NALP3 and recurrent vulvovaginal candidiasis in women with vulvar vestibulitis syndrome. Am J Obstetr Gynecol. (2009) 200:303–e1. 10.1016/j.ajog.2008.10.039 19254587

[271] Donders GGG Babula O Bellen G Linhares IM Witkin SS. Mannose-binding lectin gene polymorphism and resistance to therapy in women with recurrent vulvovaginal candidiasis. BJOG. (2008) 115:1225–31. 10.1111/j.1471-0528.2008.01830.x 18715406

[272] Wojitani MDKH de Aguiar LM Baracat EC Linhares IM. Association between mannose-binding lectin and interleukin-1 receptor antagonist gene polymorphisms and recurrent vulvovaginal candidiasis. Arch Gynecol Obstetr. (2012) 285:149–53. 10.1007/s00404-011-1920-z 21655939

[273] Giraldo PC Babula O Gonçalves AKS Linhares IM Amaral RL Ledger WJ. Mannose-binding lectin gene polymorphism, vulvovaginal candidiasis, and bacterial vaginosis. Obstetr Gynecol. (2007) 109:1123–8. 10.1097/01.AOG.0000260386.17555.a5 17470593

[274] Kalia N Singh J Sharma S Kaur M. SNPs in 3′-UTR region of MBL2 increases susceptibility to recurrent vulvovaginal infections by altering sMBL levels. Immunobiology. (2019) 224:42–9. 10.1016/j.imbio.2018.10.009 30482481

[275] Kalia N Singh J Sharma S Kaur M. Impact of SNPs interplay across the locus of MBL2, between MBL and Dectin-1 gene, on women's risk of developing recurrent vulvovaginal infections. Cell Biosci. (2019) 9:35. 10.1186/s13578-019-0300-4 31080578 PMC6505208

[276] Glocker EO Hennigs A Nabavi M Schäffer AA Woellner C Salzer U. A homozygous CARD9 mutation in a family with susceptibility to fungal infections. N Engl J Med. (2009) 361:1727–35. 10.1056/NEJMoa0810719 19864672 PMC2793117

[277] Drummond RA Brown GD. Signalling C-type lectins in antimicrobial immunity. PLoS Pathog. (2013) 9:e1003417.e1–e3. 10.1371/journal.ppat.1003417 23935480 PMC3723563

[278] Heyl KA Klassert TE Heinrich A Müller MM Klaile E Dienemann H. Dectin-1 is expressed in human lung and mediates the proinflammatory immune response to nontypeable Haemophilus influenzae. mBio. (2014) 5:e01492-14.e1-e9. 10.1128/mBio.01492-14 25161190 PMC4173778

[279] Valdimarsson H. Infusion of plasma-derived mannan-binding lectin (MBL) into MBL-deficient humans. Biochem Soc Trans. (2003) 31:768–9. 10.1042/bst0310768 12887300

[280] Valdimarsson H Vikingsdottir T Bang P Saevarsdottir S Gudjonsson JE Oskarsson O. Human plasma-derived mannose-binding lectin: a phase I safety and pharmacokinetic study. Scand J Immunol. (2004) 59:97–102. 10.1111/j.0300-9475.2004.01357.x 14723627

[281] Bang P Laursen I Thornberg K Schierbeck J Nielsen B Valdimarsson H. The pharmacokinetic profile of plasma-derived mannan-binding lectin in healthy adult volunteers and patients with Staphylococcus aureus septicaemia. Scand J Infect Dis. (2008) 40:44–8. 10.1080/00365540701522959 17852940

[282] Summerfield JA Clinical potential of mannose-binding lectin-replacement therapy. Biochem Soc Trans. (2003) 2003:770–3. 10.1042/bst0310770 12887301

[283] Yee C. Adoptive T cell therapy: points to consider. Curr Opin Immunol. (2018) 51:197–203. 10.1016/j.coi.2018.04.007 29730057

[284] Pelfrene E Mura M Sanches AC Cavaleri M Monoclonal antibodies as anti-infective products: a promising future? Clin Microbiol Infect. (2018) 2018:24 10.1016/j.cmi.2018.04.024 PMC7128139 29715552

